# Growth Behavior of Selected *Ustilaginaceae* Fungi Used for Mannosylerythritol Lipid (MEL) Biosurfactant Production – Evaluation of a Defined Culture Medium

**DOI:** 10.3389/fbioe.2020.555280

**Published:** 2020-10-21

**Authors:** Alexander Beck, Susanne Zibek

**Affiliations:** ^1^Institute of Interfacial Process Engineering and Plasma Technology IGVP, University of Stuttgart, Stuttgart, Germany; ^2^Fraunhofer Institute for Interfacial Engineering and Biotechnology IGB, Stuttgart, Germany

**Keywords:** *Ustilaginaceae*, mannosylerythritol lipids, growth curves, small-scale fermentation, oxygen online measurement, defined medium, mineral medium

## Abstract

Fungi of the *Ustilaginaceae* family are a promising source for many biotechnologically relevant products. Among these, mannosylerythritol lipid (MEL) biosurfactants have drawn a special interested over the last decades due to their manifold application possibilities. Nevertheless, there is still a knowledge gap regarding process engineering of MEL production. As an example, no reports on the use of a chemically defined culture medium have been published yet, although such a defined medium might be beneficial for scaling-up the production process toward industrial scale. Our aim therefore was to find a mineral medium that allows fast biomass growth and does not negatively affect the successive MEL production from plant oils. The results showed comparable growth performance between the newly evaluated mineral medium and the established yeast extract medium for all seven investigated *Ustilaginaceae* species. Final biomass concentrations and specific growth rates of 0.16-0.25 h^–1^ were similar for the two media. Oxygen demand was generally higher in the mineral medium than in the yeast extract medium. It was shown that high concentrations of vitamins and trace elements were necessary to support the growth. Increasing starting concentrations of the media by a factor of 10 resulted in proportionally increasing final biomass concentrations and up to 2.3-times higher maximum growth rates for all species. However, it could also lead to oxygen limitation and stagnant growth rates when too high medium concentrations were used, which was observed for *Ustilago siamensis* and *Moesziomyces aphidis*. Successive MEL production from rapeseed oil was effectively shown for 4 out of 7 organisms when the mineral medium was used for cell growth, and it was even enhanced for two organisms, *M. aphidis* and *Pseudozyma hubeiensis pro tem.*, as compared to the established yeast extract medium. Conversion of rapeseed oil into MEL was generally improved when higher biomass concentrations were achieved during the initial growth phase, indicating a positive relationship between biomass concentration and MEL production. Overall, this is the first report on the use of a chemically defined mineral medium for the cell growth of *Ustilaginaceae* fungi and successive MEL production from rapeseed oil, as an alternative to the commonly employed yeast extract medium.

## Introduction

Microorganisms of the *Ustilaginaceae* family are regarded as a promising source for many biotechnologically relevant value-added products. These basidiomycetous fungi, which include plant-pathogenic smuts as well as non-pathogenic yeasts, have been shown to produce several enzymes, organic acids, carbohydrates, lipids and biosurfactants that might be of commercial interest (Paulino et al., 2017). Among those, mannosylerythritol lipid (MEL) biosurfactants have aroused a special interested over the last decades. They show for example interesting self-assembling properties and phase-behavior (Worakitkanchanakul et al., 2009; Fukuoka et al., 2012), cell-differentiation activity (Isoda et al., 1997) and interaction with proteins (Konishi et al., 2007). They have also been reported to be valuable ingredients for hair and skin care products (Morita et al., 2010; Yamamoto et al., 2012), for agro-chemicals (Fukuoka et al., 2015), and they could be used as surface-modifiers in bioplastics (Fukuoka et al., 2018).

**FIGURE 1:**
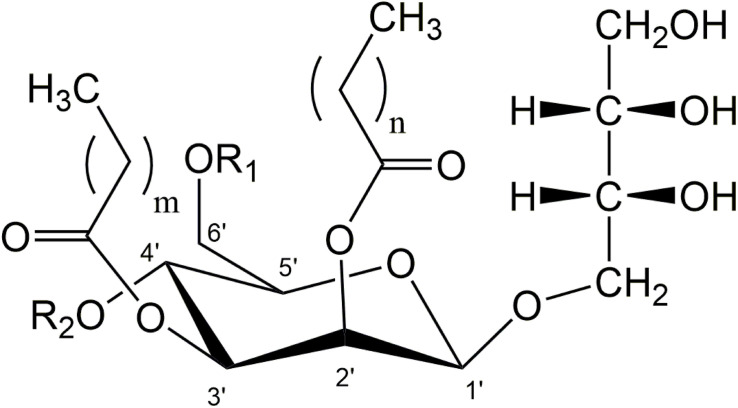
General structure of di-acylated mannosylerythritol lipids (MEL). A different degree of acetylation at C4′ and C6′ position of mannose leads to the variants MEL-A, -B, -C, and -D (MEL-A: R_1_ = R_2_ = Ac; MEL-B: R_1_ = Ac, R_2_ = H; MEL-C: R_1_ = H, R_2_ = Ac, MEL-D: R_1_ = R_2_ = H). Variable chain-length and saturation of fatty acid side-chains can be found at C2′ and C3′ (*m* = 2–16, *n* = 2–10).

MEL consist of a hydrophilic sugar part, 4-*O*-β-D-mannopyranosyl-D-erythritol, and a hydrophobic tail usually comprised of two fatty acid chains with individual chain length esterified at C2′ and C3′ of the mannose (Figure 1). At C4′ and C6′, a variable degree of acetylation is observed, leading to the distinction of classical variants MEL-A, -B, -C, -D (Kitamoto et al., 1990). The di-acetylated MEL-A is the most hydrophobic variant, followed by the mono-acetylated MEL-B and MEL-C, while the non-acetylated MEL-D is the most hydrophilic congener. The individual chain length of the two fatty acid residues is mainly influenced by the respective producer organism, as we have recently shown in a detailed study (Beck et al., 2019a). Besides, some minor co-products are produced under certain conditions like the mono- or tri-acylated MELs (Fukuoka et al., 2007) or so-called mannosylmannitol lipids (MML) (Morita et al., 2009).

Many studies on MEL production in shake flasks have already been published, identifying novel producer strains, characterizing their respective MEL product and assessing basic cultivation parameters like temperature or pH, as summarized in several reviews (Paulino et al., 2016; Saika et al., 2018; Beck et al., 2019b). Culture media have also been studied to a certain extent, mainly with regard to different carbon and nitrogen sources (Kitamoto et al., 1990; Rau et al., 2005b). The production process of MEL commonly employs a hydrophobic carbon source. Plant oils like soybean, sunflower or rapeseed oil are among the most frequently used substrates (Beck et al., 2019b). Sugars or glycerol can be used additionally or sequentially to enhance biomass growth and/or MEL production.

Surprisingly, all publications dealing with MEL production employ a complex growth medium for cell growth of the *Ustilaginaceae*, usually containing yeast extract as a source for vitamins, trace elements and also additional nitrogen compounds. Until today, there are no reports on the use of a defined mineral medium for the growth of these MEL production strains, although such defined media might be beneficial for scaling-up the production process toward industrial scale (Zhang and Greasham, 1999). Despite being readily available, widely used in lab scale and usually showing a good growth performance, yeast extract media have some drawbacks like their undefined composition, lot-to-lot variability, sensitivity to sterilization and therefore potential inhibiting components that become increasingly important when transferring the process to industrial production scale. Moreover, complex media also require increased inspection, quality control and process validation, thus adding to the cost of the overall process (Posch et al., 2012). In defined mineral media in contrast, individual components like phosphates, vitamins and trace elements can be tailored exactly to meet the physiological demand of the microorganisms, which is not possible with undefined extracts from, e.g., hydrolyzed yeast. As such, mineral media can lead to a higher process consistency and allow for better process control. Although it is quite hard to generally state the difference in cost between complex and defined mineral media, cost reduction is only possible with defined media, since they can be optimized by using well-established approaches like elementary balancing, chemostat cultures, simulation approaches or genetic algorithms (Zhang and Greasham, 1999).

Another issue that is especially relevant in biosurfactant processes is foaming (Winterburn and Martin, 2012). Foaming is caused by surface-active agents, of which the biosurfactant products and fatty acid substrates for example are the main drivers, but it can also be influenced to a significant degree by proteins that are present in the medium. Hence, when employing a yeast extract medium, its protein content might also add to foaming issues in those processes.

Besides, there is still a general knowledge gap regarding the detailed process engineering of MEL production. For example, there are only few studies regarding characterization of growth parameters of *Ustilaginaceae* fungi like growth rates, substrate consumption, biomass yield or oxygen requirements, just to name a few. It would also be interesting to get a deeper insight into how those growth parameters and the resulting biomass concentration are related to successive MEL production. Moreover, bioreactor processes have only been published for a few *Ustilaginaceae* species. The most frequently cited publication regarding fermentation of MEL in a bioreactor is the work by Rau et al. (2005a), whereby *M. aphidis* was used in a fed-batch process with glucose and soybean oil as main carbon sources, sodium nitrate as inorganic nitrogen source and yeast extract as a complex source for organic nitrogen, vitamins and trace elements. After the cease of initial growth on glucose, nitrate and yeast extract at about 24 h of cultivation, a concentrated solution of the three components was fed to further increase biomass concentration. A small amount of soybean oil (2% v/v) was initially used to reduce foaming. Further oil addition was then triggered by the antifoam sensor when foaming was strong, leading to an additional feed of 20% v/v oil (related to initial reactor volume). It was claimed that the increased biomass, resulting from increased substrate feeding, together with the additional oil feed led to improved MEL concentrations. However, despite the oil feeding – as well as reducing aeration and stirring during production phase – the whole process was characterized by enormous foaming, which caused a significant amount of broth to leave the reactor, presumably via the off-gas, and disturbed the cultivation process (Rau et al., 2005a). Such a behavior might still be handled on a manual lab-scale but not in an automated industrial process.

Due to some of the drawbacks when using yeast extract media for cell growth, the replacement of complex ingredients by defined minerals and vitamins could be advantageous. The aim of this study therefore was to find a mineral medium that allows comparable growth of the fungi, does not negatively affect the successive MEL production from rapeseed oil, and to derive key parameters like growth and substrate consumption rates or biomass yields. For this, we characterized the batch growth behavior of seven different *Ustilaginaceae* fungi, which we had previously investigated for their MEL structures (Beck et al., 2019a), in a micro-bioreactor system. The microbioreactor system can be used to efficiently speed up bioprocess development in an early stage without the necessity to perform multiple bench-top fermentations or shake flask experiments, as nicely shown by Wewetzer et al. (2015). We screened and evaluated three different culture media at six different concentrations mainly with regard to biomass formation, pH and oxygen requirements, but also with relation to subsequent MEL production from rapeseed oil. The first medium was the frequently employed complex yeast extract medium for MEL production (Rau et al., 2005a). The second was a chemically defined growth medium with salts, vitamins and trace elements that was developed for Saccharomyces cultivation (Theobald et al., 1993) and that we now transferred for the cultivation of *Ustilaginaceae* fungi. The third medium was a defined minimal medium with lower concentrations of vitamins and trace elements. A detailed comparison of the respective compositions is presented in the materials and also discussion section. In all three media we used glucose and sodium nitrate as the major carbon and inorganic nitrogen source for cell growth. Rapeseed oil was then used as the main substrate to induce the MEL production phase after the cease of growth according to Günther et al. (2015). Overall, this study provides valuable insights into the growth and MEL production behavior of those fungi, which will help us and other researchers to design and scale-up bioreactor production processes with defined and optimized mineral media in the future.

## Materials and Methods

### Microorganisms

Seven fungal microorganisms of the *Ustilaginaceae* family that were previously identified as potential MEL producers (Beck et al., 2019a) were used for the growth characterization study. *M. aphidis* DSM 70725 was obtained from the German Collection of Microorganisms and Cell Cultures GmbH (DSMZ; Braunschweig, Germany). *Moesziomyces parantarcticus* CBS 10005, *Pseudozyma hubeiensis pro tem*. CBS 10077, *Pseudozyma tsukubaensis pro tem*. CBS 422.96, *Sporisorium graminicola* CBS 10092, *Ustilago siamensis* CBS 9960 and *Ustilago shanxiensis* CBS 10075 were obtained from the Westerdijk Fungal Biodiversity Institute (CBS-KNAW; Utrecht, Netherlands). These seven strains were selected from the larger variety of MEL producer species since they represent the variety of different genera within the *Ustilaginaceae* family.

### Chemicals and Culture Media

Chemicals used throughout this work were obtained from Merck (previously Sigma-Aldrich, Darmstadt, Germany), Carl Roth (Karlsruhe, Germany) or Th. Geyer (Renningen, Germany) unless noted otherwise. Deionized water was used to prepare the culture media.

Three different liquid culture media were employed for seed cultures and for subsequent experiments in the microtiter-cultivation system and shaking flasks (Table 1). Medium 1 was a complex yeast extract medium based on a formula according to Rau et al. (2005a) with 1 g L^–1^ yeast extract (Gibco Bacto, Thermo Fisher Scientific), initial pH 6 (not adjusted). Medium 2 was a mineral medium after Theobald et al. (1993) with defined salt, vitamin and trace element solutions, initial pH 5.5 (not adjusted). In the trace element solution, concentrations of CuSO_4_ and MnSO_4_ were reduced to half the concentration of Theobald et al. (1993) according to previous results of our group (not published). Medium 3 was based on the yeast nitrogen base without amino acids and ammonium sulfate (YNB Y1251, Merck) formula, with lower concentrations of vitamins and trace elements than medium 2. The detailed composition for all three media is presented in Table 1 and will be discussed in the results section.

**TABLE 1:** Composition of the three media evaluated within this study.

Medium Type Reference	1 Complex Rau et al. (2005a)	2 Mineral Theobald et al. (1993)	3 Mineral YNB	
Glucose	30.0	30.0	30.0	g L^–1^
NaNO_3_	3.0	3.0	3.0	g L^–1^
CaCl_2_	–	0.1	0.1	g L^–1^
KCl	–	1.1	–	g L^–1^
KH_2_PO_4_	0.2	1.0	1.0	g L^–1^
MgSO_4_	0.1	0.5	0.5	g L^–1^
NaCl	–	–	0.1	g L^–1^
Yeast extract	1	–	–	g L^–1^
CuSO_4_	–	0.77	0.04	mg L^–1^
FeCl_3_	–	9.00	0.20	mg L^–1^
MnSO_4_	–	4.73	0.40	mg L^–1^
ZnSO_4_	–	5.05	0.40	mg L^–1^
H_3_BO_3_	–	–	0.50	mg L^–1^
Na_2_MoO_4_	–	–	0.20	mg L^–1^
KI	–	–	0.10	mg L^–1^
Biotin	–	0.03	0.002	mg L^–1^
Ca-Pantothenate	–	30.0	0.4	mg L^–1^
Myo-Inositol	–	60.3	2	mg L^–1^
Pyridoxin HCl	–	1.5	0.4	mg L^–1^
Thiamin HCl	–	6.0	0.4	mg L^–1^
Folic acid	–	–	0.002	mg L^–1^
Niacin	–	–	0.4	mg L^–1^
p-aminobenzoic acid	–	–	0.2	mg L^–1^
Riboflavin	–	–	0.2	mg L^–1^

*For reasons of comparability, the salts and trace elements are all listed in anhydrous form, although they might actually be added in the hydrate form.*

In order to study the influence of medium concentration on maximum biomass, growth rates and oxygen limitation, additional dilution levels of 1:1.5, 1:2, 1:3, 1:6 and 1:10 (equivalent to glucose concentrations of 20, 15, 10, 5, and 3 g L^–1^) were prepared from those three stock media by dilution with deionized water. By doing so, only the absolute concentration of the ingredients was reduced while the relative composition of all elements remained constant.

Raffinated rapeseed oil (K-classic, Kaufland Warenhandel GmbH & Co. KG, Germany) was used as the main carbon substrate for MEL synthesis during production phase. It was autoclaved and added to the microtiter plates under sterile conditions after the cease of cellular growth.

### Seed Culture Preparation

Cryo cultures of the microorganisms were kept at −80°C in glycerol and thawed only once for immediate use. Stock cultures were plated on potato dextrose (PD) agar plates and incubated at 30°C for 3 days. Seed culture was performed in two steps in 15-mL tubes (6 mL working volume, polypropylene caps) and 100-mL baffled shaking flasks. The flasks were always filled with 20 vol-% culture medium and sealed with BIO-SILICO sterile stoppers (Hirschmann Laborgeräte GmbH & Co. KG, Germany) to ensure sufficient oxygen supply. For the seed cultures, the same culture media as in the main experiment were used. The first seed culture was inoculated with two full loops from the agar slants and incubated for 4 h at 200 rpm (shaking diameter *d* = 50 mm) and 30°C to disperse cells. The second seed culture was inoculated with 1–2 mL of the first seed culture to a defined optical density (OD_625_) of 0.05 and incubated for 24 h (medium 1) or 48 h (medium 2 and 3) at 110 rpm (shaking diameter *d* = 50 mm) and 30°C, until a minimum OD_625_ of 3 was reached. Incubation time for the seed cultures in medium 2 and 3 had to be prolonged due to a longer lag phase of the cells compared to the medium 1 culture.

### Microtiter Cultivation and *Online*-Analysis to Assess Growth, pH- and O_2_-Requirements

For cultivation experiments, the 48-well microtiter “Flowerplates” (MTP-48-BOH, m2p Labs GmbH, Baesweiler, Germany) sealed with adhesive gas-permeable and evaporation-reduced membranes (F-GPR48-10, m2p Labs GmbH, Baesweiler, Germany) were used. Online-characterization of growth behavior was performed with the BioLector I (m2p Labs GmbH, Baesweiler, Germany) micro-fermentation system. Non-invasive online measurements included scattered light (so-called “backscatter,” which is directly related to biomass concentration) as well as pH and dissolved oxygen (DO) through so-called *optodes* at the bottom of each well. Gain values of 20 and 5 were used for backscatter measurements in growth and production phase, respectively. Calibration of pH- and DO-optodes is done by the manufacturer for each batch of microtiter plates.

**FIGURE 2:**
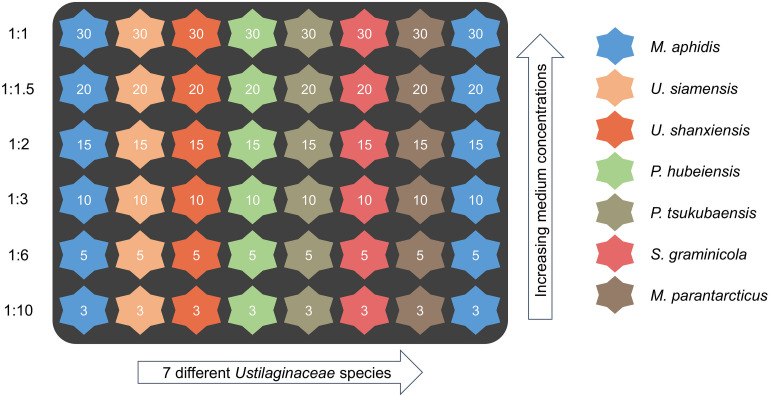
Microtiter cultivation plate layout for evaluation of microorganisms and initial medium concentrations (dilution levels). The numbers in the wells indicate initial concentrations of glucose (g L^–1^) in the medium, which was used as the reference compound. The identical cultivation scheme was applied for all three media. A duplicate row of *M. aphidis* on every plate was used to assess the relative errors of measurement.

The wells of the microtiter plates were filled with 1,000 μL of culture medium according to the scheme shown in Figure 2. For each of the three investigated media, a single plate with this layout was used to evaluate all seven species and different starting concentrations of the respective medium. A duplicate row of *M. aphidis* was used to determine the relative errors within the plates. Inoculation volume was adjusted to yield an initial OD_625_ of 0.3 (corresponding to an average backscatter of 20 a.u. at gain 20) for each microorganism. Volume increase by inoculation was ensured to be below 10%, thus not changing the hydrodynamics of the system significantly. Temperature and humidity in the system were controlled at 30°C and 85% relative humidity and the plates were agitated at 1,100 rpm with a shaking diameter of *d* = 3.0 mm. Cycle time was set at 10 min, allowing for sequential measurement of backscatter at gain 5 and 20, pH and DO every 10 min. The specific growth rates μ during biomass growth were calculated from backscatter gain 20. A moving average of backscatter data (30 measuring intervals) was used to reduce noise. In order to determine the specific oxygen demand of the microorganisms during growth and MEL production, actual oxygen uptake rates (OUR_act_) were approximated from the measured dissolved oxygen levels (DO_act_) in the BioLector. For the FlowerPlates, a technical maximum OTR_max,tech_ of 50 mmol L^–1^ h^–1^ is stated by the manufacturer for conditions of 1,000 μL filling volume and 1,100 rpm shaking speed. This equals a volumetric mass transfer coefficient (*k*_L_a value) of 198 h^–1^ at 30°C and ambient pressure of 1 bar. Such values are also commonly observed in benchtop bioreactors at lab-scale, showing the good transferability of the results between the microcultivation system and bioreactors. The actual OUR_act_ during microbial cultivation in the microtiter plates was hence calculated from the measured dissolved oxygen level DO_act_ [%] using following formula [see also Wewetzer et al. (2015)]:

$$OUR_{act}=k_{L}a\times H_{O2}\times p_{O2}\times \left(DO_{max}-DO_{act}\right)/100$$

where *k*_L_a is the volumetric mass transfer coefficient for selected shaking conditions (198 h^–1^); H_O2_ is the Henry constant for oxygen in water at 30°C (1.2 mmol L^–1^ bar^–1^) and p_O2_ is the oxygen partial pressure in the gas phase (0.21 bar). Due to the controlled environment, it was assumed that all coefficients remain constant during the experiment.

After the cease of growth, MEL production was initiated by addition of 80 μL (8% v/v) rapeseed oil to each well of the microtiter plate in order to investigate the influence of the different media on the successive MEL production from rapeseed oil. The respective times for oil addition were 72 h for medium 1 and 48 h for medium 2 and 3. In the results part, no online data is shown for the production phase, as the oil addition had an influence on backscatter and DO measurements. Data for pH during the production phase showed interesting insights and will be discussed later.

### Parallel Analysis of Substrate Consumption in Shaking Flasks

Since the microliter-volumes of cultivations in 48-well microtiter plates do not allow for extended sampling, additional shaking flask cultivations were performed to assess substrate consumption of the microorganisms and ensure transferability of the results [see also Wewetzer et al. (2015)]. This was done for all seven species with medium 1 and 2, at initial medium concentrations of 30 g L^–1^ glucose and 3 g L^–1^ NaNO_3_ (undiluted condition) respectively. For this, 1-L shaking flasks with three baffles were filled with 200 mL of the respective media and inoculated to an initial OD_625_ of 0.3 like in the microtiter plate cultivations. The flasks were incubated at 110 rpm (shaking diameter *d* = 50 mm) and 30°C. All cultivations in shaking flasks were performed in triplicates. Samples were taken at *t* = 0, 4, 20, 24, 28, 44, 48, 52, and 70 h to measure glucose and NaNO_3_ concentrations as well as OD_625_. The growth phase lasted for either 70 h (medium 1) or 48 h (medium 2). At this time, dry biomass was measured to determine biomass yields from glucose and nitrate, respectively. MEL production was then also initiated by addition of 16 mL (8% v/v) rapeseed oil similar to the experiments in the microcultivation system.

For the determination of glucose and nitrate concentration, 1 mL samples were centrifuged for 10 min at 16,000 *g* and the supernatant collected. For glucose analysis, the supernatant was diluted with an appropriate volume of 5 mM H_2_SO_4_, filtered with 0.2 μm cellulose acetate filters and measured by HPLC using a SUPELCOGEL 8H column (30 cm × 7.8 mm, 9 μm, Sigma-Aldrich, Darmstadt, Germany) and 5 mM H_2_SO_4_ as mobile phase at a flow rate of 0.6 mL/min and 30°C. For nitrate measurements, the supernatant was diluted with de-ionized water and analyzed with an enzymatic nitrate test kit (R-Biopharm, Darmstadt, Germany). For the measurement of dry biomass, samples of 5 mL were pipetted onto pre-dried and weighed filters and washed twice with 0.9% w/v NaCl and ethanol to remove possible contaminations. After drying at 110°C overnight, the filters were weighed again and dry biomass was calculated.

Calculation of volumetric and specific consumption rates as well as biomass related yields for glucose and nitrate was done according to the following formula:

$$r_{\text{S}}=\frac{\Delta c_{\text{S}}}{\Delta \text{t}};q_{\text{S}}=\frac{1}{c_{\text{x}}}\frac{\Delta c_{\text{S}}}{\Delta t};Y_{X,S}=\frac{\Delta c_{\text{x}}}{\Delta c_{\text{S}}}$$

Where *r*_S_ [g L^–1^ h^–1^] is the volumetric consumption rate, *q*_S_ [g g^–1^ h^–1^] the biomass specific consumption rate, *Y*_X/S_ the biomass related yield [g g^–1^], and *c*_S_ and *c*_X_ the concentrations of substrates and biomass, respectively. The index *S* denotes substrate, i.e., glucose or sodium nitrate, and index *X* denotes biomass. Substrate consumption rates were calculated for each time interval and the maximum value evaluated. Biomass yields were calculated as an average from the final biomass and total amount of consumed substrate. Respective errors are given as standard deviation from the triplicates.

### Extraction and HPTLC Quantification of MEL

For quantification of residual plant oil (acylglycerides), released fatty acids and the produced MEL at the end of the cultivation (*t* = 175 h), 800 μl of culture sample was taken from each well of the 48-well microtiter plates and mixed with an equal amount of ethyl acetate. Extraction was performed by shaking at 1,500 rpm and 30°C for 10 min, followed by centrifugation at 16,000 *g* and room temperature for 5 min to separate the two phases and the cells. Then, 500 μL of the organic supernatant was separated and the solvent evaporated. The extract was finally weighed, re-suspended in ethanol to match the calibration range and analyzed via high-performance thin layer chromatography (HPTLC).

HPTLC was performed on HPTLC silica 60 plates (20 mm × 10 mm, Merck, Germany) using a solvent system consisting of chloroform-methanol (20:3, v/v). Samples were spotted with an ATS4 automatic TLC sampler (CAMAG, Muttenz, Switzerland). After development, the HPTLC plates were stained by dipping for 1 s into an acetic acid/*p*-anisaldehyde/sulfuric acid (97:1:2 v/v/v) reagent solution, heated and quantified densitometrically with the gel analyzer function of ImageJ. With this HPTLC method, a discrimination between plant oil (triacylglycerides, TAG), free fatty acids (FFA) and the different MEL variants MEL-A,-B,-C, and -D is possible. Identification and calibration of MEL concentration was done with a representative purified MEL standard from the respective microorganism. MEL standards were produced and analyzed as published previously (Beck et al., 2019a). Fatty acid and residual oil concentrations were calibrated using oleic acid and rapeseed oil as standards.

## Results and Discussion

Growth of the seven *Ustilaginaceae* species was investigated with three different medium formulations, all containing glucose as the main carbon source. Sodium nitrate was always used as inorganic nitrogen source in all media. Stock solutions of all three media were formulated with 30 g L^–1^ glucose and 3 g L^–1^ NaNO_3_ similar to Rau et al. (2005a), which is equal to a molar C/N ratio of 28.4 mol_C_/mol_N_. Such a comparatively high *C*/*N* ratio usually leads to nitrogen limitation and therefore triggers the secondary lipid metabolism, as it has been observed for many oleaginous yeasts (Beopoulos et al., 2011). This is also a favorable condition for the production of glycolipid biosurfactants (Jezierska et al., 2017). Concentrations of phosphate, sulfate and the macro-elements Mg^2+^ and Ca^2+^ were similar between medium 2 and 3 and higher than in the medium 1 with yeast extract. The main difference between medium 2 and 3 was the concentration of vitamins and trace elements (see Table 1). In medium 2, the final concentrations of all vitamins and trace elements were approximately 10- to 50-fold higher than in medium 3. For example, concentration of Fe^3+^ ions, which are an important co-factor for enzymes and which we identified as a potential inducer for the MEL transporter (Beck and Zibek, 2020), was 45-times higher. In medium 1, no additional vitamins or trace elements were added as they should already be contained in the yeast extract. Yeast extract also contains additional amino acids or short peptides, which were not present nor supplemented in the other two media. Consequently, all three media had the same glucose and nitrate concentrations but differed in the amount of mineral salts, vitamins or trace elements, which is important for the following interpretation and discussion of the results.

### Mineral Medium Shows Comparable Maximum Biomass Concentrations and Growth Rates

**FIGURE 3:**
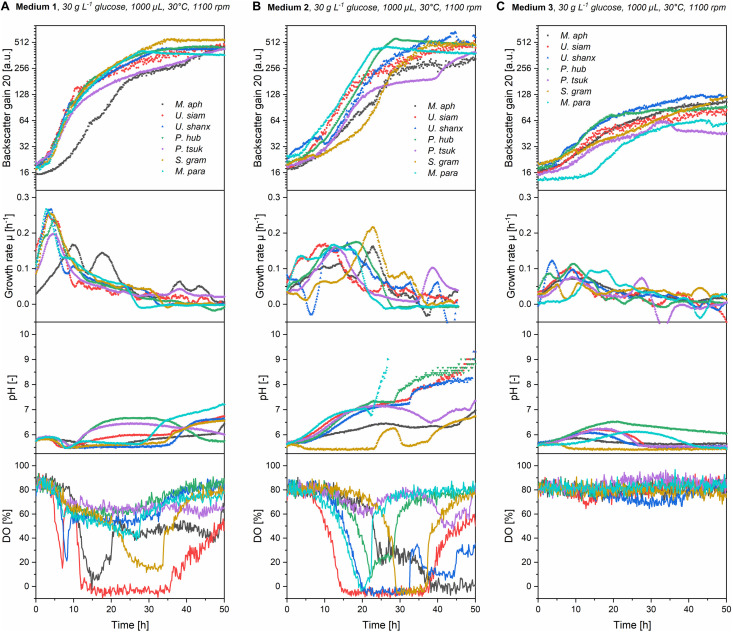
Online growth profiles (backscatter, growth rates, pH and DO) for all seven *Ustilaginaceae* species in medium 1 **(A)**, medium 2 **(B)**, and medium 3 **(C)** with an initial concentration of 30 g L^–1^ glucose equivalent. Growth rates in medium 1 were high initially but then decreased fast. In medium 2, they were lower at the beginning but maintained over a longer time. Stationary phase was thus reached faster in medium 2. In medium 2, a stronger pH drift and higher oxygen consumption, resulting in low DO values, were generally observed.

**FIGURE 4:**
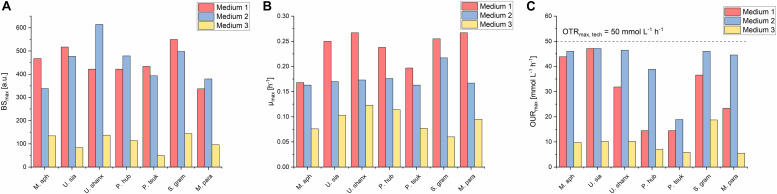
Comparison of maximum final backscatter BS_max_ **(A)**, maximum specific growth rates μ_max_ **(B)**, and maximum oxygen uptake rates OUR_max_ **(C)** for all seven *Ustilaginaceae* species in medium 1-3 at concentrations of 30 g L^–1^ glucose equivalent. BS_max_ values were comparable for medium 1 and 2, but much lower in medium 3. Medium 1 had the highest μ_max_. Oxygen demand was highest in medium 2.

A comparison of the three media regarding their growth profiles (Figure 3) as well as the resulting maximum backscatter BS_max_ [correlates to biomass and/or optical density, see for example Back et al. (2016)] and maximum specific growth rates μ_max_ (Figure 4) showed that the mineral medium 2 yielded comparable biomass growth to the complex medium 1 for all seven species, while there was a significantly lesser growth in medium 3. The BS_max_ values for medium 2 were in the same range as for medium 1. For some species like *U. shanxiensis*, *P. hubeiensis pro tem.* and *M. parantarcticus* they were even higher in medium 2. Medium 3 in turn showed much lower BS_max_ for all seven species.

The highest specific growth rates were generally achieved with medium 1, with μ_max_ values between 0.17 and 0.25 h^–1^. This corresponds to a cell-doubling time of *t*_D_ = 2.8–4.1 h. Medium 2 yielded slightly lower μ_max_ of 0.16–0.21 h^–1^ (*t*_D_ = 3.3–4.3 h) for all seven species. The μ_max_ values of our *Ustilaginaceae* species correspond with values reported by Faria et al. (2014). Assessing different hexoses and pentoses as carbon sources, also without yeast extract supplementation and using yeast nitrogen base without amino acids (YNB Y0626, Difco), for growth of *M. antarcticus*, *M. aphidis* and *M. rugulosus* in shaking flasks, this group found max. specific growth rates between 0.13 and 0.25 h^–1^, depending on the species and sugar used (Faria et al., 2014). Although there is also variation in their data, the general range of growth rates is similar to the values reported here.

By looking at the kinetics of growth in Figure 3 and the characteristic times where maximum backscatter and growth rates were obtained (*t*_BS,max_ and *t*_μ_,_max_ in Table 2), it becomes clear that the high maximum growth rates in medium 1 are observed within the first hours of growth. This is presumably due to the complex ingredients of the medium that are contained in the yeast extract and do not have to be synthesized *de novo* by the organisms, like for example amino acids. After an initial growth phase of about 5–10 h, the growth slows down considerably and enters a second growth phase on the inorganic nitrate. In medium 2 in contrast, all essential metabolites like amino acids need to be synthesized from the inorganic nitrogen source, thus requiring a longer time until the full growth speed is achieved. At the same time, the maximum growth rates in the mineral medium were subsequently sustained over a longer time, mostly until the substrates were exhausted. As an effect, only a single growth phase is observed in medium 2 and the stationary phase is reached even faster than in medium 1 (*t*_BS,max_ in Table 2). To support this point, we also looked at the substrate consumption in those two media in additional shake flask experiments (see below).

**TABLE 2:** Characteristic times for maximum backscatter (*t*_BS,max_), maximum growth rates (*t*_μ_,_max_), and minimum DO level (*t*_DO,min_) in the three media (medium 1–3) for all seven *Ustilaginaceae* species.

	*t*_BS,max_ [h]			*t*_μ,max_ [h]			*t*_DO,min_ [h]		
Species/medium	1	2	3	1	2	3	1	2	3
*M. aphidis*	≥50*	≥50*	≥50*	9.7	22.9	12.7	14.9	43.4	n.s.^#^
*U. siamensis*	≥50*	≥50*	48.2	3.3	10.2	8.7	17.7	17.0	n.s. ^#^
*U. shanxiensis*	34.7	37.5	≥50*	4.0	16.0	3.7	8.2	23.0	n.s. ^#^
*P. hubeiensis*	46.9	29.0	≥50*	4.8	18.3	9.2	15.5	22.2	n.s. ^#^
*P. tsukubaensis*	≥50*	≥50*	32.4	4.3	13.2	9.2	43.4	44.2	n.s. ^#^
*S. graminicola*	36.7	37.9	≥50*	3.7	22.7	10.7	29.4	29.5	n.s. ^#^
*M. parantarcticus*	28.4	26.4	≥50*	2.7	12.3	14.2	26.7	19.7	n.s. ^#^

*Max. backscatter was not clearly reached within the investigated time, # no significant change in DO level observed.

Medium 3 yielded the lowest growth rates with μ_max_ values of 0.06–0.12 h^–1^ (*t*_D_ = 5.8–11.6 h) and only over a short time period, which corresponds to the low BS_max_ values obtained. Hence, medium 3 was not as balanced as medium 2 for efficient biomass growth, presumably due to the 10- to 50- times lower amounts of vitamins and trace elements. In a separate experiment with a modified medium 2, where we removed all of the vitamins and/or trace elements (not published), a significant reduction of growth rates and resulting biomass concentrations was observed, supporting this hypothesis. Addition of other vitamins and trace elements to medium 2 did not show significant differences (not published), indicating that the current vitamin and trace element solution of medium 2 is well balanced for the requirements of *Ustilaginaceae* fungi.

### Differences in Substrate Consumption Between the Media

**FIGURE 5:**
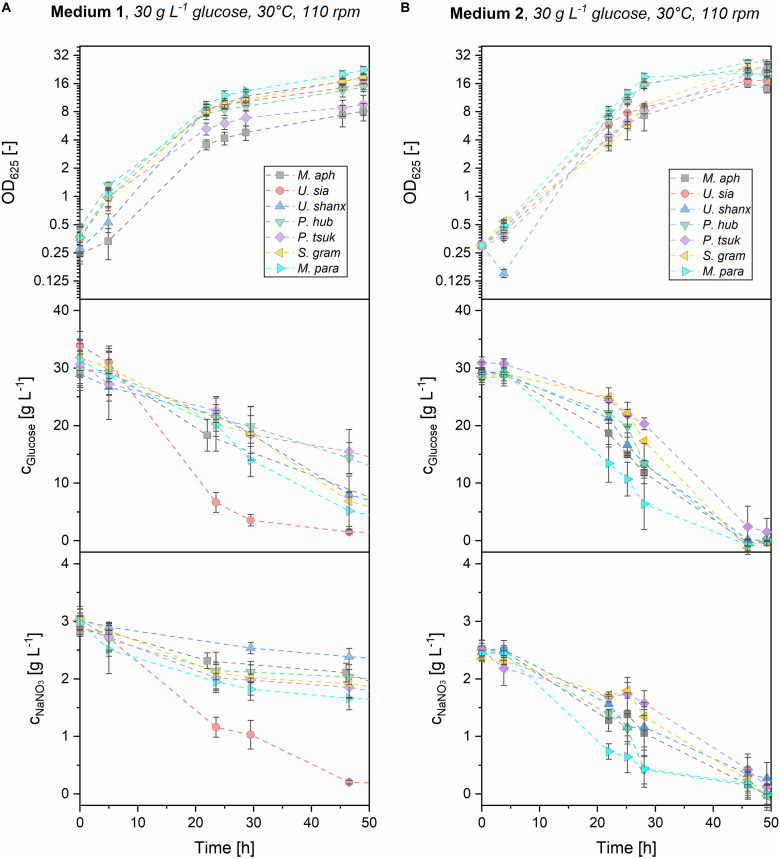
Offline analysis of growth and substrate consumption (OD_625_, glucose and NaNO_3_) for all seven *Ustilaginaceae* species in medium 1 **(A)** and medium 2 **(B)** with initial substrate concentrations of 30 g L^–1^ glucose and 3 g L^–1^ NaNO_3_. Both substrates were efficiently consumed in medium 2 within 48 h. In medium 1, glucose and nitrate were still unconsumed after 48 h, most probably due to other organic nitrogen sources present in the yeast extract, which are consumed first.

To get a better understanding of the differences in growth between the yeast extract medium 1 and the mineral medium 2, consumption of the main substrates glucose and sodium nitrate was investigated. For this, parallel shaking flask experiments were performed where the substrate concentrations were measured over time. OD was also measured to ensure that the growth profiles were similar between the microtiter plates and the shaking flasks. Medium 3 was not investigated here, as the overall growth and substrate consumption were too low. Figure 5 shows the kinetics of growth and substrate consumption for medium 1 and 2 in the shake flasks.

In medium 2, both glucose and nitrate were consumed from the beginning and were fully exhausted after 48 h for all seven microorganisms. *M. parantarcticus*, *U. shanxiensis*, and *P. hubeiensis pro tem.* showed the fastest growth together with high substrate consumption rates, while *P. tsukubaensis pro tem.* had the slowest consumption rates. This coincides with the findings from the microtiter cultivations, where the same trends were observed. There is also a good correlation of OD measurements from shaking flasks and the respective backscatter values obtained at the same cultivation time points (see Supplementary Figure 1). Transferability of the results between the systems was thus verified.

Values for maximum glucose and nitrate consumption rates in medium 2 were in the range of *r*_Gluc,max_ = 1.0–2.2 g L^–1^ h^–1^ and *r*_Nit,max_ = 0.089–0.244 g L^–1^ h^–1^, respectively (Table 3). Hence, glucose consumption rates in medium 2 are approximately 10 times higher than nitrate consumption rates in all seven species. At the current ratio of glucose-to-nitrate, this results in a simultaneous exhaustion and thus limitation of both substrates when the stationary phase is entered, which might be favorable for the successive MEL production on plant oils. To determine the average biomass yields, the final resulting biomass *c*_x,final_ was measured as dry biomass and divided by the consumed substrate until this time. Average biomass yields from glucose *Y*_X/Gluc,av_ = 0.12–0.23 g g^–1^ and nitrate *Y*_X/Nit,av_ = 1.52–2.64 g g^–1^ were obtained (see Table 3). It is interesting to note that *M. aphidis* produced a high amount of biomass despite the low OD_625_ and backscatter values, which can be reasoned by its hyphal morphology (discussed below). As a result, *M. aphidis* was the organism that achieved the highest biomass yields from glucose and nitrate with values of *Y*_X/Gluc_ = 0.23 ± 0.06 g g^–1^ and *Y*_X/Nit_ = 2.64 ± 0.99 g g^–1^, respectively, leading to a biomass concentration of 6.9 ± 1.3 g L^–1^ from 30 g L^–1^ glucose and 3 g L^–1^ nitrate. These values correspond again with those from Faria et al. (2014), where a biomass concentration of 8.7 ± 2.4 g L^–1^ and a yield of 0.27 ± 0.04 g g^–1^ from 40 g L^–1^ glucose had been reported for *M. aphidis*.

**TABLE 3:** Substrate consumption rates and dry biomass related yields for glucose and sodium nitrate in medium 2 for the seven *Ustilaginaceae* species.

Species	*r*_gluc,max (0–48__h)_ [g L^–1^ h^–1^]	*r*_nit,max (0–48 h)_ [g L^–1^ h^–1^]	*q*_gluc,max (0–48 h)_ [g g^–1^ h^–1^]	*q*_nit,max (0–48 h)_ [g g^–1^ h^–1^]	*c*_x,final_ [g L^–1^]	*Y*_X/Gluc,av (0–48 h)_ [g g^–1^]	*Y*_X/Nit,av (0–48 h)_ [g g^–1^]
*M. aphidis*	1.14 ± 0.06	0.112 ± 0.021	0.50 ± 0.03	0.059 ± 0.011	6.86 ± 1.30	0.23 ± 0.06	2.64 ± 0.99
*U. siamensis*	–	0.152 ± 0.005	–	0.114 ± 0.004	2.29 ± 0.17	–	1.28 ± 0.04
*U. shanxiensis*	1.45 ± 0.13	0.124 ± 0.007	0.74 ± 0.07	0.065 ± 0.004	5.12 ± 0.38	0.17 ± 0.03	2.30 ± 0.31
*P. hubeiensis*	2.22 ± 0.13	0.244 ± 0.049	0.84 ± 0.05	0.092 ± 0.019	5.59 ± 0.31	0.20 ± 0.02	2.19 ± 0.57
*P. tsukubaensis*	1.00 ± 0.06	0.089 ± 0.009	0.81 ± 0.05	0.063 ± 0.007	3.68 ± 0.17	0.12 ± 0.01	1.52 ± 0.23
*S. graminicola*	1.65 ± 0.03	0.154 ± 0.010	0.85 ± 0.01	0.079 ± 0.005	5.55 ± 0.08	0.19 ± 0.01	2.29 ± 0.17
*M. parantarcticus*	1.48 ± 0.39	0.095 ± 0.026	0.96 ± 0.26	0.103 ± 0.029	4.01 ± 0.26	0.14 ± 0.05	1.61 ± 0.55

*Cultivations were performed as independent triplicates in shaking flasks.*

In medium 1, substrate consumption was significantly different to medium 2. Due to the additional carbon and nitrogen sources originating from the yeast extract that could not be quantified, a calculation of consumption rates and biomass yields were not performed for medium 1. However, the qualitative time course of glucose and nitrate concentrations (Figure 5) will be discussed, as there are significant differences to medium 2. After 48 h, there was still residual glucose and nitrate left for all species. Full exhaustion of glucose was observed after about 72 h for five of the organisms (not shown), i.e., 24 h later than in medium 2. For *P. tsukubaensis pro tem.* and *P. hubeiensis pro tem.*, there was even a significant concentration of 5–10 g L^–1^ glucose remaining after 72 h. Sodium nitrate concentrations after 48 h were still at levels of 1.5–2.3 g L^–1^ for all species, indicating a much lower consumption of inorganic nitrogen source in medium 1 compared to medium 2. It can be assumed that the lower consumption of nitrate results from the preferred consumption of other nitrogen sources that are present in the yeast extract of medium 1. Differential nitrogen consumption and its complex genetic regulation has been shown for the related species *U. maydis* (Horst et al., 2012). According to the supplier, yeast extract contains about 10% w/w elementary nitrogen, which is equivalent to an additional elementary nitrogen concentration of 0.1 g L^–1^ from yeast extract in medium 1. Compared with the 0.5 g L^–1^ elementary nitrogen that come from NaNO_3_, it is quite significant. These additional nitrogen components in yeast extract, such as amino acids, nucleotides and peptides, could be used almost directly for the synthesis of proteins and DNA, thus enabling a faster growth especially at the start of cultivation. This is in accordance with the high growth rates in the first 5–10 h of growth for all microorganisms on medium 1. After initial consumption of the organic nitrogen sources in the yeast extract, the cells switch their metabolism to consumption of nitrate, which is more difficult to assimilate and thus leads to lower growth rates in this second growth phase. In order to get nitrogen limitation, the initial concentration of inorganic nitrate would have to be reduced in medium 1.

*U. siamensis* showed a clear difference regarding substrate consumption in medium 1 when compared with the other six species. This strain exhibited high glucose and nitrate consumption, but at the same time did not produce more biomass than other strains. This could be attributed to the generation of extracellular mucus, most likely polysaccharides, which made the culture broth very viscous. This phenomenon has also been described before by Sajna et al. (2013a), who observed the production of 3.5 g/L extracellular polysaccharide with the strain *Pseudozyma sp.* NII 08165 after 4 days under similar conditions. The polysaccharide consisted of glucose, galactose and mannose and had a molecular weight of 1.7 MDa. Sajna et al. (2013b) later identified the strain *Pseudozyma sp.* NII 08165, which also produced MELs, as phylogenetically closest to *U. siamensis*.

In the future, the substrate consumption rates and yields for the seven organisms with medium 2 can be used to determine the stoichiometry for biomass growth and to model the (batch) growth kinetics, which will then serve as a base for developing a more robust fed-batch process with this medium.

### Mineral Medium Leads to Higher Oxygen Demand and a Stronger pH Drift Than Complex Medium

As already mentioned in the introduction, no detailed information on pH or oxygen requirements for the seven *Ustilaginaceae* species was available in literature. Nevertheless, both pH and dissolved oxygen are important control parameters in a bioreactor process. Here, cultivation in the microtiter cultivation system allowed for a non-invasive and continuous online-measurement of both pH and DO during cultivation, which is a great advantage over shaking flasks for example. The trends of pH and DO during growth phase, presented in Figure 3, could be linked to the increase in biomass and respective growth rates and might be used for an enhanced process control in the future.

In medium 1, which is buffered by the yeast extract, pH remained between 5.5 and 7 during growth phase. After a short drop of the pH to 5.5 during the initial growth phase between 5 and 10 h, pH values stayed mostly constant or increased to around pH 7 during the second phase. In medium 2 in contrast, pH values mostly showed a steadily increasing trend from the beginning of growth, before they increased again sharply after the cells reached the stationary phase. Overall, strongly alkaline pH values were observed toward the end of growth in medium 2, indicating a low buffering capacity of the present medium.

Dissolved oxygen levels in medium 1 decreased to around 50–60% within the first 10 h of growth for all species. Afterward, DO levels remained constant for four of the organisms. Only *U. siamensis*, *M. aphidis*, and *S. graminicola* showed a further decrease of DO values and thus the highest oxygen demand OUR_max_ in medium 1 (Figure 4). *U. siamensis* even entered a phase of complete oxygen limitation between 12 and 35 h. Oxygen limitation occurred when OUR_max_ was equivalent to the maximum oxygen transfer rate of the system (OTR_max,tech_) of 50 mmol L^–1^ h^–1^. As it can be expected, DO values increased again once the cells reached their stationary phase. The trends of pH and DO correspond well with the measured growth rates in medium 1. During the initial growth on the yeast extract ingredients at high growth rates, pH and DO decreased fast. During the second growth phase on the inorganic nitrogen source at lower growth rates, they remained more or less constant.

Oxygen demand OUR_max_ in medium 2 was higher than in medium 1 for all species (Figure 4). DO levels in medium 2 decreased sharply when the cells were growing at their maximum growth rates, which were usually reached at 10–20 h. As a result, this resulted in the respective minimum DO values between 17 and 29 h for five species (*t*_DO,min_ in Table 2) and could even lead to full oxygen limitation, which lasted until the substrates were completely consumed and the cells entered into stationary phase. It was also observed that oxygen limitation led to a direct decrease in growth rates and therefore prevented the microorganisms from faster growth.

The lowest oxygen demand, in both media 1 and 2, was measured for *P. hubeiensis pro tem.* and *P. tsukubaensis pro tem.*, where no oxygen limitation was observed at the respective media concentrations. *U. siamensis* in turn showed the highest oxygen demand and longest phase of oxygen limitation. The low DO values for this strain during growth phase in medium 1 and 2 presumably result from the production of extracellular polysaccharide, as mentioned before. This leads on the one hand to an increased oxygen demand for the production and on the other hand to a higher viscosity in the culture medium, thus impeding with mixing and oxygen transfer. This can pose a specific challenge when designing a bioreactor process for this species, as it has been reported for production of other microbial extracellular polymers, for example in xanthan gum fermentations (Rosalam and England, 2006).

In medium 3, levels of DO and pH did show almost no alteration over the cultivation time, which corresponds to the low growth rates and low biomass in this medium. As a result, no oxygen limitation or pH shift occurred.

Overall, we could observe that the mineral medium 2 resulted in a higher demand of oxygen and also led to a more alkaline pH after the cease of growth for all species. Oxygen limitation resulted in decreasing growth rates and consequently prevented even faster growth in this medium. For a future bioreactor process with medium 2, we propose that an active pH control should be realized that prevents the alkaline drift and that can be adjusted to the individual optimum for growth and MEL production of each microorganism. Additionally, a regulation of DO level in the bioreactor, e.g., by dynamic adaptation of the stirring speed or air flow rate, should be employed to prevent oxygen limitation and thus the decrease of growth rates, which has been observed for most of the species in medium 2. This approach is for example different to Rau et al. (2005a) or Goossens et al. (2016), where pH and DO were not controlled.

### Increasing Medium Concentrations Lead to Higher Final Biomass and Growth Rates but Also to an Increasing Oxygen Demand

As the next step, we used dilution series of the three media to determine the relationship between initial medium concentrations and the resulting final backscatter BS_max_ and maximum specific growth rates μ_max_. Moreover, from relating OUR_max_ with medium concentration, we could determine the respective concentrations that led to oxygen limitation and study the influence of such an oxygen limitation on the growth behavior for each organism in more detail.

**FIGURE 6:**
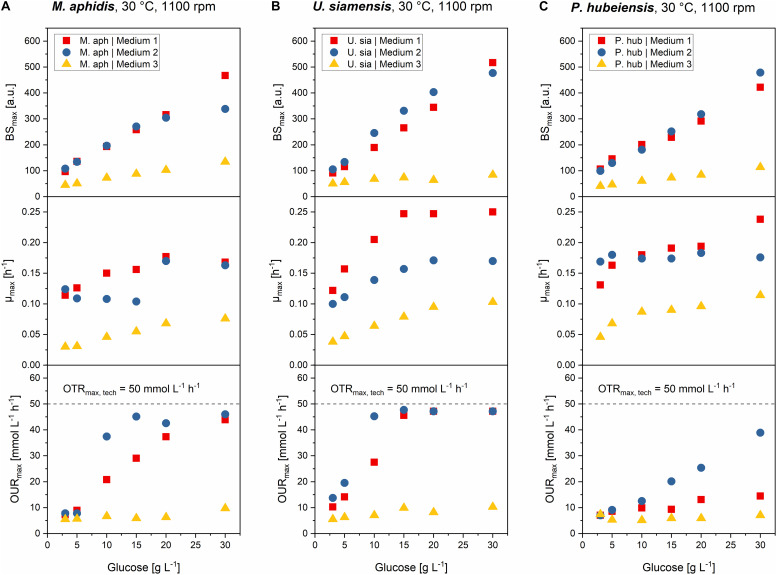
Comparison of max. final backscatter BS_max_, max. specific growth rate μ_max_ and max. oxygen uptake rates OUR_max_ for *M. aphidis* **(A)**, *U. siamensis* **(B)**, and *P. hubeiensis pro tem.* **(C)** in the three media with increasing medium concentrations from 3 to 30 g L^–1^ glucose equivalent. BS_max_ values were comparable between medium 1 and 2, and increased proportionally with medium concentration. Maximum growth rates μ_max_ increased until the point of oxygen limitation. Oxygen limitation occurred when the OUR_max_ values were equal to the OTR_max_. The respective medium concentration that led to oxygen limitation was different for each species and medium.

For this, the stocks of the three media, which were prepared with 30 g L^–1^ glucose, were diluted with deionized water to have exactly the same relative medium composition but lower concentrations of all components. In the following description, the different dilutions will be related to their glucose content of 30, 20, 15, 10, 5, and 3 g L^–1^, respectively (schematic overview see Figure 2). The results for BS_max_, μ_max_ and OUR_max_ over initial medium concentration, which is represented by the respective glucose concentration, are shown in Figure 6 for the three species *M. aphidis*, *U. siamensis*, and *P. hubeiensis pro tem.* as general examples. Results for the other four species, which showed the same trends, can be found in the Supplementary Figures 9, 10. The respective time profiles for backscatter, growth rate, pH and DO, from which those values were determined, are also included in the Supplementary Figures 2–8.

The final BS_max_ values increased proportionally with increasing medium concentration, meaning that for example a two times higher initial medium concentration led to two times higher biomass in stationary phase. Biomass yields therefore can be seen as constant over the investigated range of substrate concentrations. This was true for all seven species and all three media, with only two exceptions: For *M. aphidis* and *U. siamensis* in medium 2 with the highest initial medium concentration of 30 g L^–1^ glucose equivalent, lower-than-expected backscatter values were obtained, probably due to the strong oxygen limitation that already occurred at medium concentrations equivalent to 10–15 g L^–1^ glucose (Figure 6). Hence, the biomass yield was reduced for those two species at the highest concentration level.

The derived μ_max_ values also increased with higher initial concentrations, although not in a linear way. Usually, μ_max_ was more strongly increased in the lower range of substrate concentrations, for example between 3 and 10 g L^–1^ glucose, but remained constant with further increasing substrate concentration. The reasons for this can be seen when looking at the OUR_max_, respectively. Increasing the medium concentrations led to higher oxygen demand and at a certain point to oxygen limitation (OUR_max_ = OTR_max,tech_), which in turn resulted in a reduced growth rate. For *U. siamensis* for example, oxygen limitation occurred already at glucose levels of 10 g L^–1^. Others like *P. hubeiensis pro tem.* or *P. tsukubaensis pro tem.* did not yield an oxygen limitation at 30 g L^–1^glucose, so medium concentration could have been increased even further. As a general conclusion, respective oxygen demand, as well as the maximum medium concentration that did not lead to oxygen limitation, were significantly different for the seven *Ustilaginaceae* species.

Over the full range of investigated medium concentrations from 3 to 30 g L^–1^ glucose equivalent, μ_max_ values could be increased around 1.3- to 2.3-fold, usually as long as oxygen was not limiting. The largest increase in μ_max_ with increasing medium concentration was found for *P. tsukubaensis pro tem.* in medium 1, where the ten-fold increase in initial substrate led to a 2.3-fold increase in μ_max_. At the same time, this species did not suffer from oxygen limitation due to a very low oxygen demand, which explains this strong increase in μ_max_. For *P. hubeiensis pro tem.* in turn, no change in μ_max_ was observed in medium 2 with increasing substrate concentration, although no oxygen limitation was observed. Alterations in μ_max_ with changing medium concentration was hence species-specific and most probably depending on other limiting components in the medium besides carbon, nitrogen or oxygen. In future work, individual medium components should be optimized to increase growth rates of the microorganisms further and to achieve a faster process.

It can be concluded that an increase of initial medium concentration, in the range from 3 to 30 g L^–1^ glucose equivalent, generally resulted in linearly increased biomass formation (BS_max_) during batch growth. Only two species made an exemption here, and only at the highest concentration. Values for μ_max_ were also slightly increased with increasing initial concentrations, but only to the point until oxygen limitation occurred. Oxygen limitation led to a decrease in specific growth rates, thus μ_max_ could not be increased any further. To achieve even higher μ_max_ values or higher biomass concentration from increased amounts of glucose, an enhanced oxygen transfer for example by increasing *k*_L_a through higher stirrer speeds or higher aeration in a bioreactor would be necessary. On the other hand, a fed-batch strategy with growth rates slightly below the critical growth rates for oxygen limitation could also be designed, leading to efficient growth and higher biomass from increased amounts of glucose without limitation of oxygen supply.

### Successive MEL Production From Rapeseed Oil Is Enhanced by Higher Biomass Concentrations and Correlates With a Change in pH-Value

Finally, we investigated the process-related relationship of biomass concentration achieved during growth phase and successive MEL production by adding 8% v/v of rapeseed oil as substrate to the microtiter plates after primary growth was ceased and the cells entered their stationary phase. Induction of MEL production-related pathways by oil addition in stationary phase has previously been shown by Günther et al. (2015). Oil addition was done after 72 h for medium 1 and 48 h for medium 2 and 3, respectively. After a total 175 h of cultivation, the wells of the microtiter plates were finally extracted with ethyl acetate and the crude extract analyzed by HPTLC for residual oil, fatty acids and MEL. This was done for all seven microorganisms and all three media with the different initial concentrations.

**FIGURE 7:**
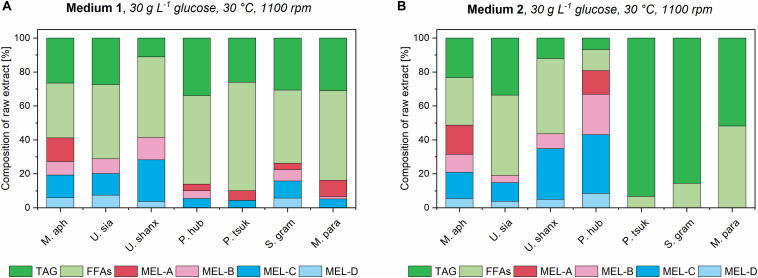
Composition of organic extracts for all seven *Ustilaginaceae* fungi in medium 1 **(A)** and medium 2 **(B)** at the highest concentration, after induction of MEL production with 8% v/v rapeseed oil. Relative amounts of unconsumed triglycerides (TAG), free fatty acids (FFAs) and the different MEL variants are shown.

All seven species showed successful MEL production with medium 1, as expected (Figure 7). MEL production in medium 3 was not detected in significant amounts, most probably due to the low biomass concentrations in this medium. With medium 2, MEL was produced in four of the seven species. From the three species that did not produce MEL in medium 2 (*S. graminicola*, *P. tsukubaensis pro tem.*, and *M. parantarcticus*), two also had low amounts of free fatty acids present in the extract, indicating a low rate of oil hydrolysis. A possible reason for this could for example be the high pH values that were obtained after the cease of growth and during the consecutive production phase in medium 2 (Supplementary Figure 11). High pH might inhibit the secretion or activity of extracellular lipases that are required for oil hydrolysis. However, it should be noted that *P. tsukubaensis pro tem.* and *M. parantarcticus* showed a low MEL production in medium 1 as well, so the low production might also be due to other parameters than culture medium composition. By verifying that no glucose was present at the end of the growth phase, we could exclude the possibility of catabolite repression, which might have been another possible reason for low oil hydrolysis.

Encouragingly, MEL production was enhanced for *M. aphidis* and *P. hubeiensis pro tem.* in the newly employed medium 2 compared to the established yeast extract medium 1. At the highest medium concentration of 30 g L^–1^ glucose, the relative percentage of total MEL in the extract could be increased from 41% in medium 1 to 49% in medium 2 for *M. aphidis* and from 14 to 80% for *P. hubeiensis pro tem.* respectively. Thus, we could see that medium 2 is already well balanced not only for growth of those two species, but also for MEL production.

**FIGURE 8:**
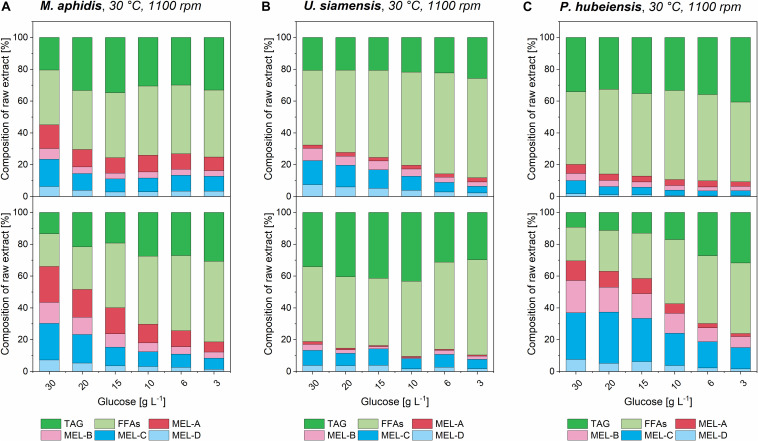
Composition of organic extracts from *M. aphidis* **(A)** *U. siamensis* **(B)**, and *P. hubeiensis pro tem.* **(C)** cultures in different concentrations of medium 1 (upper) and medium 2 (lower), after initiation of MEL production with 8% v/v rapeseed oil as substrate. Relative amounts of unconsumed triglycerides (TAG), free fatty acids (FFAs) and the different MEL variants are shown. For *M. aphidis* and *P. hubeiensis pro tem.*, a generally higher conversion from oil to MEL in the extracts was achieved with medium 2 compared to medium 1. Increasing medium concentration in growth phase, which led to higher biomass concentrations (see Figure 6), showed a positive effect on overall conversion of oil into MEL for all species, while the relative proportions of the MEL-variants MEL-A, -B, -C, -D remained constant.

By looking at the different concentration levels of medium 1 and 2 a relationship between increasing medium concentration, which resulted in a higher biomass as shown before, and the following MEL conversion could be determined. According to the general theory for product formation, for example by Luedeking and Piret (1959), a higher biomass should yield higher catalytic capacity and therefore result in a better bioconversion of plant oil into MEL. The results for the three species *M. aphidis*, *U. siamensis*, and *P. hubeiensis pro tem.* with increasing concentrations of medium 1 and 2 are shown in Figure 8. For the four other species the respective figures can be found in the Supplementary Figures 12–15. It was observed for all seven species that an increased concentration of medium, which led to higher biomass concentrations after growth (compare Figure 6), could also increase the conversion of triglycerides and fatty acids into MEL. However, the relationship was not always proportional, which is similar to the results for the growth rates and might be due to the observed oxygen limitation or other limiting factors. Nevertheless, we could verify that MEL product formation can be positively related with biomass concentration obtained at the end of the growth phase. A similar result had been presented by Kitamoto et al. (1992a), where increasing concentration of resting cells of *M. antarcticus*, another MEL producing species, led to higher MEL production from soybean oil. In their experimental setup, the production of MEL from 8% v/v soybean oil was almost linearly increased up to 40 g/L MEL for resting cell concentrations between 5 and 24 g/L biomass (Kitamoto et al., 1992a). For the design of a prospective bioreactor process, this is another proof that a controlled fed-batch process resulting in high biomass concentrations without oxygen limitation should be beneficial for the successive MEL production.

The relative amounts of the different MEL congeners (MEL-A, -B, -C, -D) for each species, which we had previously investigated in detail by our HPTLC-MALDI-TOF-MS method (Beck et al., 2019a), remained unchanged with variation in substrate concentration or media. This again showed that they are mainly governed by the species itself and only to a minor extend by process conditions.

Monitoring of online pH values during MEL production provided some additional interesting results. We observed that pH, which was usually at pH 7 or higher after growth phase, dropped to values of around 5.5 when oil was hydrolyzed (Supplementary Figure 11). In contrast, no oil hydrolysis and thus no MEL production was observed when pH did not drop to this value, as it was the case for *S. graminicola* and *P. tsukubaensis pro tem.* in medium 2. From the chemical point of view, the change in pH to acidic values is caused by the release of free fatty acids (pKa values of around 5) as the result of oil hydrolysis by lipase activity, and not directly related to MEL production. However, as the release of fatty acids is a prerequisite for MEL production, it can be a rate-limiting step in the MEL production process. As such, the time course of pH is a valuable monitoring tool in the bioprocess. Since we could link the drop in pH to oil hydrolysis and consecutive MEL production, these results could also be used for an enhanced process control. We hypothesize that the regulated addition of correcting agents under active pH control should correlate with the amount of released fatty acids from hydrolysis. Based on the consumption of correcting agents, the beginning of oil hydrolysis and sequential MEL production could be tracked and further oil addition triggered to enhance the production in a bioreactor. This would be an alternative strategy to the already mentioned oil feeding triggered by antifoam sensor (Rau et al., 2005a), which can lead to excessive oil feeding due to strong foaming.

### Cellular Morphology Is Not Influenced by Medium Composition

**FIGURE 9:**
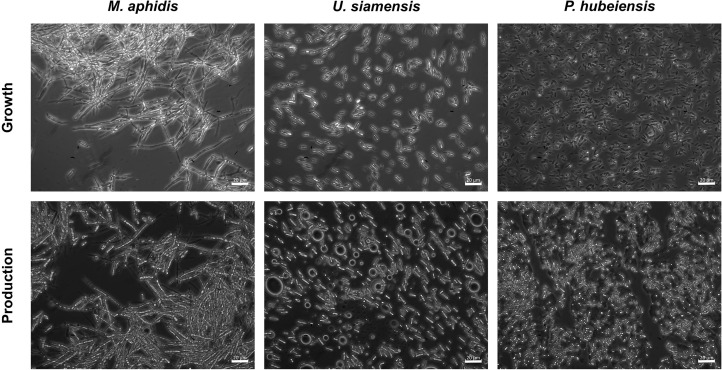
Microscope images of *M. aphidis*, *U. siamensis*, and *P. hubeiensis pro tem.* showing the difference in morphology. *M. aphidis* grew as elongated, hyphal cells that also formed large aggregations, while *U. siamensis* and *P. hubeiensis pro tem.*, as well as the other four investigated organisms, showed unicellular yeast-like growth.

Despite the differences in growth kinetics, cellular morphology of the respective organisms was unchanged when using complex, mineral or YNB medium. *M. aphidis* was the only species that grew in a hyphal or elongated form, while the other six species had a unicellular, ellipsoidal yeast-like growth (Figure 9). The hyphal cells of *M. aphidis* influenced the measurement of backscatter and OD_625_. Compared to other species, lower backscatter values were obtained for this species, although biomass concentration was the highest, as mentioned before. Therefore, the low backscatter and OD_625_ values for *M. aphidis* were not due to low cell density, but attributed to its filamentous cell shape that has an influence on optical cell density measurements. From the duplicate rows of *M. aphidis* in the microcultivation system we were able to determine the average error of biomass quantification by backscatter, which was below 5% relative error. Since *M. aphidis* with its hyphal morphology generally presents the largest challenge for biomass quantification in classical ways like OD or dry biomass, this was another proof for the soundness of results obtained with the microcultivation system. It can be assumed that relative errors for other species will be in the same range.

During the MEL production phase, i.e., after the addition of the plant oil, oil inclusion bodies were visible for all seven species, showing their ability to use triacylglycerols and MEL as storage material (Kitamoto et al., 1992b). Moreover, *M. aphidis* gradually changed its morphology to more ellipsoidal cells after oil addition. This dimorphic behavior is well known for species of the *Ustilaginaceae* family (Wang et al., 2015) and has been described before for *M. aphidis* (Rau et al., 2005b).

## Conclusion

With the results presented in this study, we have performed a detailed screening study for comparative growth characterization of the seven *Ustilaginaceae* species in an established complex and two different mineral media and derived some valuable indications and key parameters for successful cultivation and MEL production.

First, we showed that mineral medium 2 achieved similar final biomass concentrations and comparable specific growth rates to medium 1 for all species, proving its ability to be used for growth of the *Ustilaginaceae* fungi. For some species like *U. shanxiensis*, *P. hubeiensis*, and *M. parantarcticus*, biomass formation was even enhanced. From the comparison of medium 2 and 3, we could deduce that all seven investigated organisms require a high amount of vitamins and trace elements to achieve high cell densities. As these components were present in significantly lesser amounts in medium 3, a slower growth and lower final cell densities were achieved with medium 3 compared to medium 2.

The difference in growth and substrate consumption rates between the medium 1 and 2 could be linked to organic nitrogen compounds that are present in the yeast extract of medium 1 and that can be metabolized quickly. This led to two distinct growth phases in medium 1: a first phase with high growth rates on the complex ingredients, followed by a second phase on glucose and the inorganic nitrate with much lower growth rates. In medium 2, glucose and nitrate are the only carbon and nitrogen sources for growth and were therefore metabolized completely in a single growth phase with more or less constant specific growth rates. The growth and substrate consumption rates as well as the biomass yields that were determined for medium 2 can now be used for modeling the stoichiometry and kinetics of growth.

By increasing initial medium concentrations between 3 and 30 g L^–1^ glucose equivalent, we could show a proportional correlation between initial concentrations of culture medium and the resulting biomass after growth. Specific growth rates were also increased slightly for all species. We could also determine the critical growth rates where oxygen limitation is occurring, which could be used now to design a controlled fed-batch process with adapted growth rates producing high biomass concentrations without oxygen limitation. This is especially interesting because lastly we demonstrated that increasing the biomass concentration during growth can lead to a better bioconversion of plant oil into MEL in the successive production phase. In the newly employed mineral medium 2, MEL production was shown for four of the seven species and even enhanced for two of them, *M. aphidis* and *P. hubeiensis pro tem.*, when compared with the previously employed yeast extract medium.

The knowledge generated in this study can now be used to further enhance the mineral medium 2 by adjusting individual components like trace elements or vitamins for each species and to design and model an efficient fed-batch process for MEL production in a bioreactor. First results with the novel mineral medium 2 and selected species in our bioreactor have already shown good growth and MEL production performance as well as reduced foam formation, which led to a stable and controllable process. Ultimately, using higher amounts of substrate to produce more biomass within a controlled fed-batch process should be the way to go when aiming for higher MEL yields.

## Data Availability Statement

All datasets generated for this study are included in the article/Supplementary Material.

## Author Contributions

AB designed and conducted the experiments, analyzed the data, and wrote the first draft of the manuscript. SZ acquired the grants and supervised the work of AB. SZ and AB revised the manuscript, read and approved the final submitted version. All authors contributed to the article and approved the submitted version.

## Conflict of Interest

The authors declare that the research was conducted in the absence of any commercial or financial relationships that could be construed as a potential conflict of interest.
