# Supplementary material for: Growth Behavior of Selected Ustilaginaceae Fungi Used for Mannosylerythritol Lipid (MEL) Biosurfactant Production – Evaluation of a Defined Culture Medium
Source: Front Bioeng Biotechnol. 2020 Oct 21;8:555280. doi: 10.3389/fbioe.2020.555280 (PMC7609910; doi:10.3389/fbioe.2020.555280)
Supplement: Supplementary file 1 [file Data_Sheet_1.pdf]

frontiers

Supplementary Material

1 Supplementary Figures

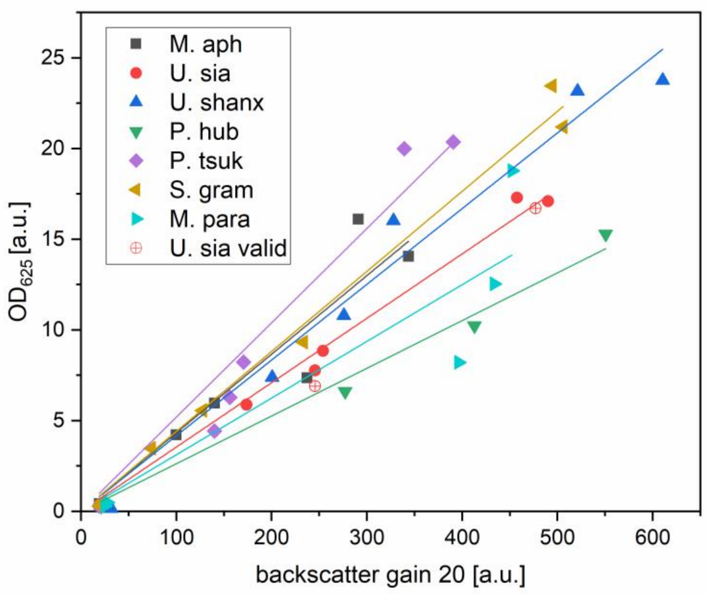

Supplementary Figure 1 Correlation of backscatter (gain 20) data from the micro-fermentation system with respective OD_{625} values from parallel shake flask cultivations for selected species in medium 2. Positive correlation of data proves the transferability of results obtained with the two methods.

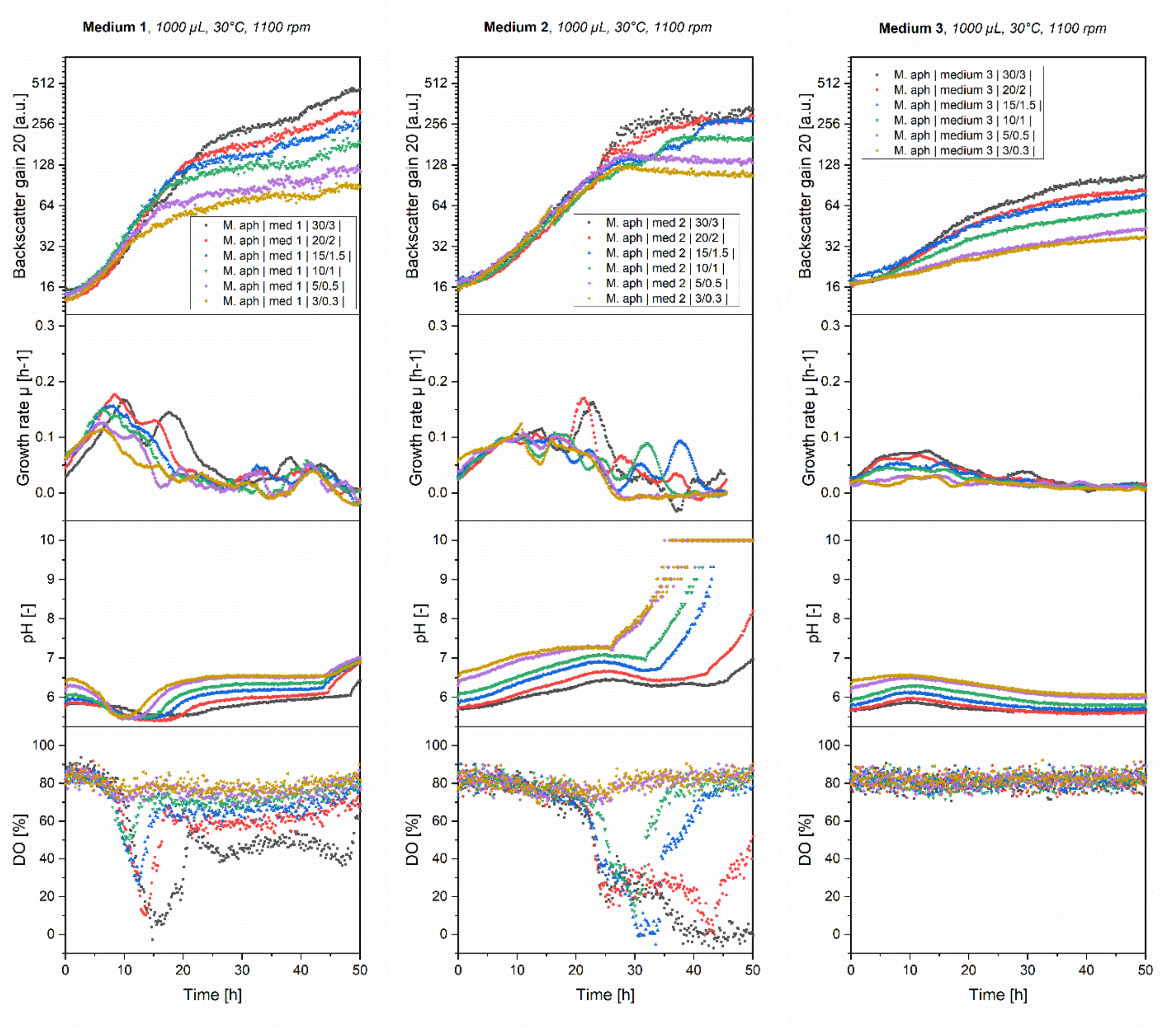

Supplementary Figure 2 Online growth profiles (backscatter, growth rates, pH and DO) for *M. aphidis* in medium 1 (left), medium 2 (middle) and medium 3 (right) with varying medium concentrations from 3 to 30 g L^{-1} glucose. Higher substrate concentrations led to higher final backscatter, slightly increasing growth rates and lower dissolved oxygen levels, i.e. higher oxygen demand.

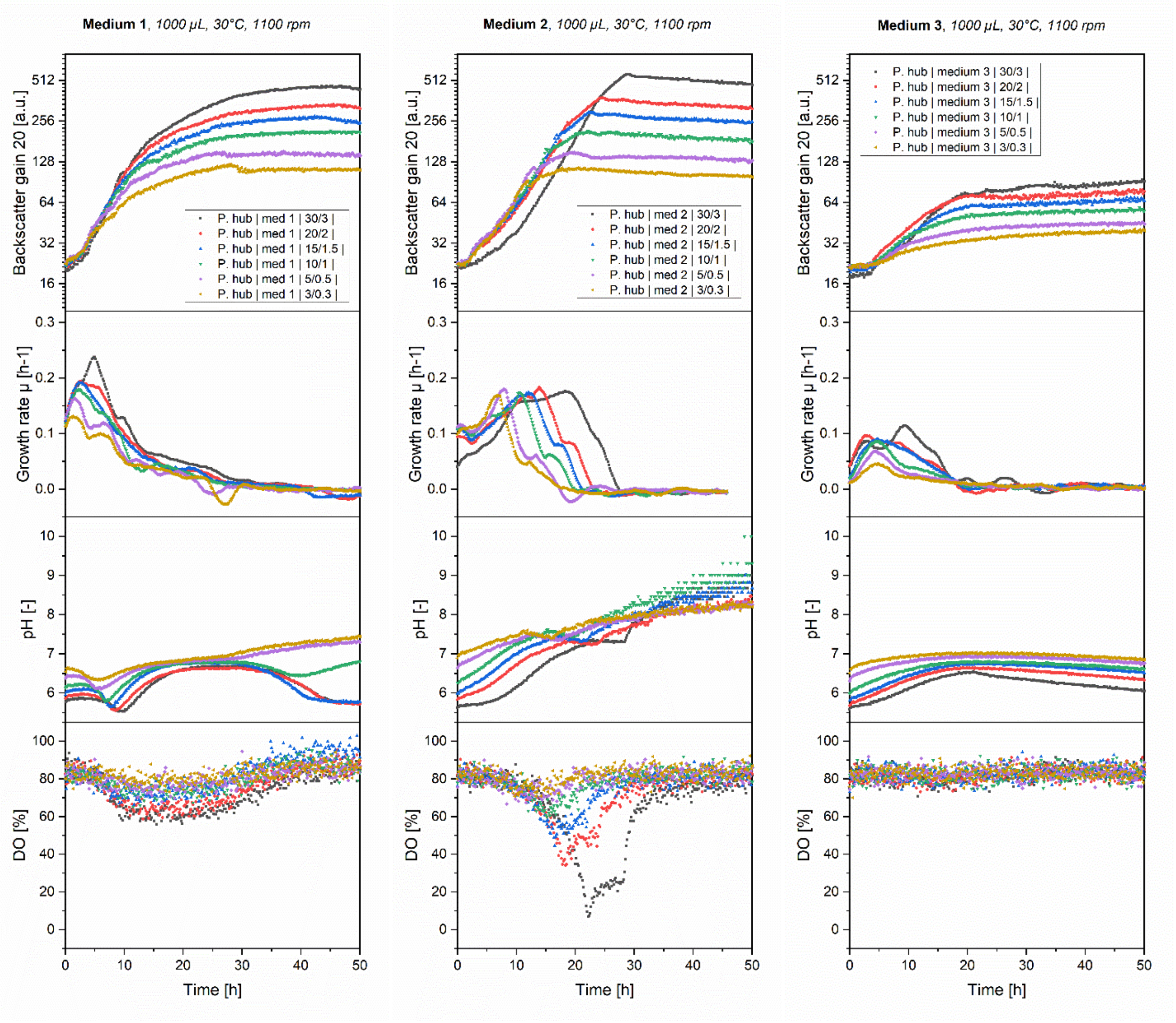

Supplementary Figure 3 Online growth profiles (backscatter, growth rates, pH and DO) for *P. hubeiensis* in medium 1 (left), medium 2 (middle) and medium 3 (right) with varying medium concentrations from 3 to 30 g L^{-1} glucose. Higher substrate concentrations led to higher final backscatter, slightly increasing growth rates and lower dissolved oxygen levels, i.e. higher oxygen demand.

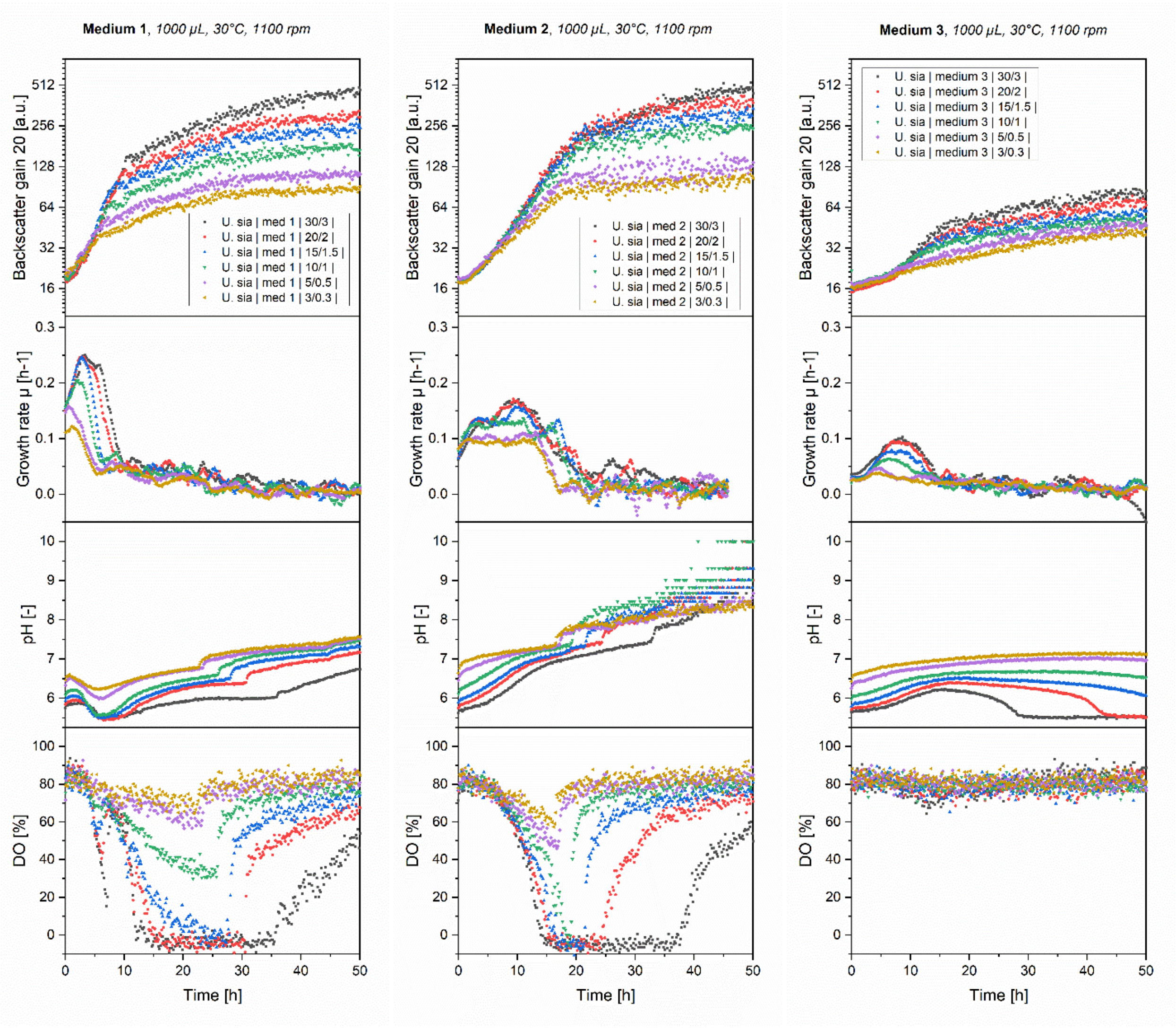

Supplementary Figure 4 Online growth profiles (backscatter, growth rates, pH and DO) for *U. siamensis* in medium 1 (left), medium 2 (middle) and medium 3 (right) with varying medium concentrations from 3 to 30 g L^{-1} glucose. Higher substrate concentrations led to higher final backscatter, slightly increasing growth rates and lower dissolved oxygen levels, i.e. higher oxygen demand.

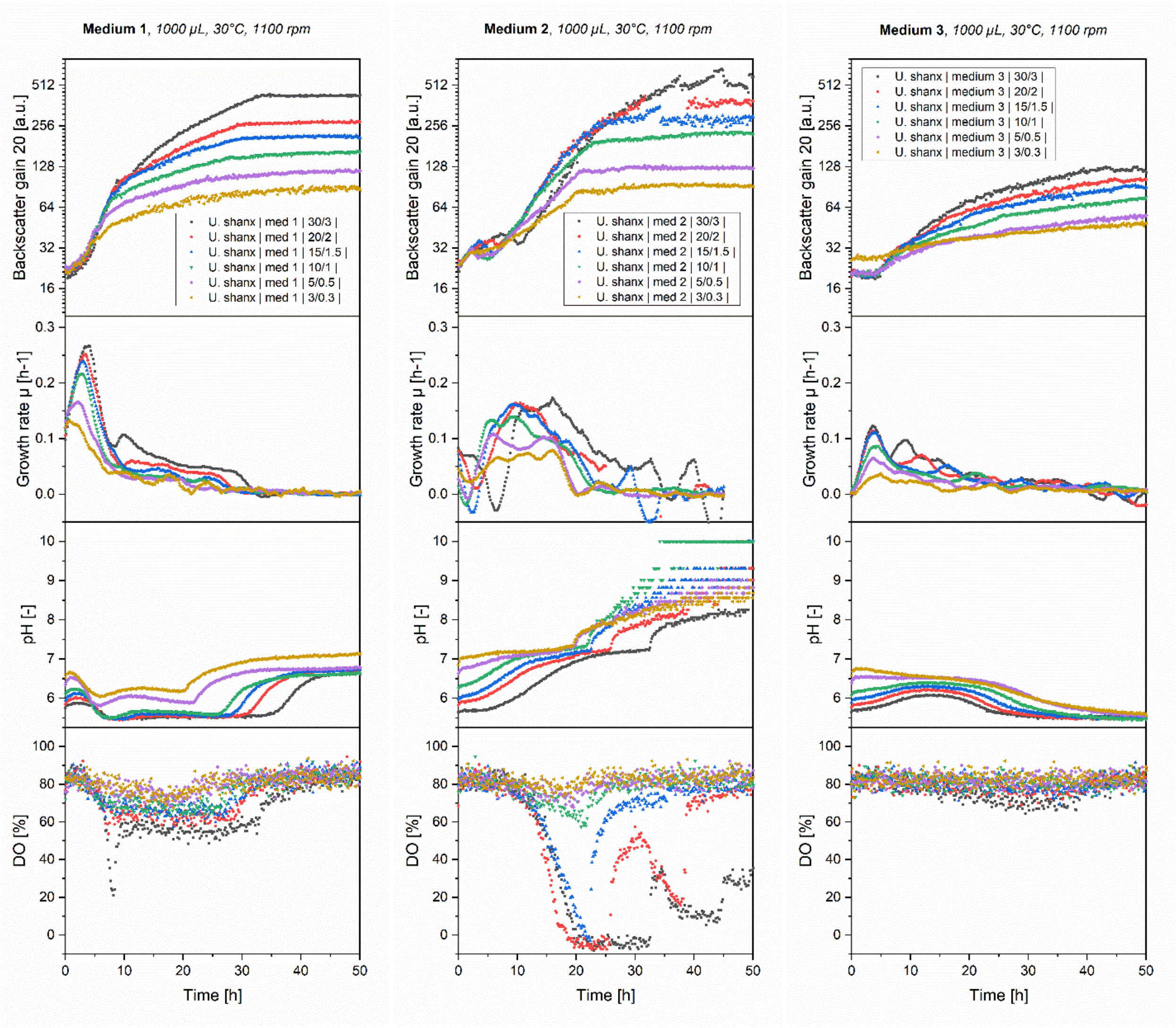

Supplementary Figure 5 Online growth profiles (backscatter, growth rates, pH and DO) for *U. shanxiensis* in medium 1 (left), medium 2 (middle) and medium 3 (right) with varying medium concentrations from 3 to 30 g L^{-1} glucose. Higher substrate concentrations led to higher final backscatter, slightly increasing growth rates and lower dissolved oxygen levels, i.e. higher oxygen demand.

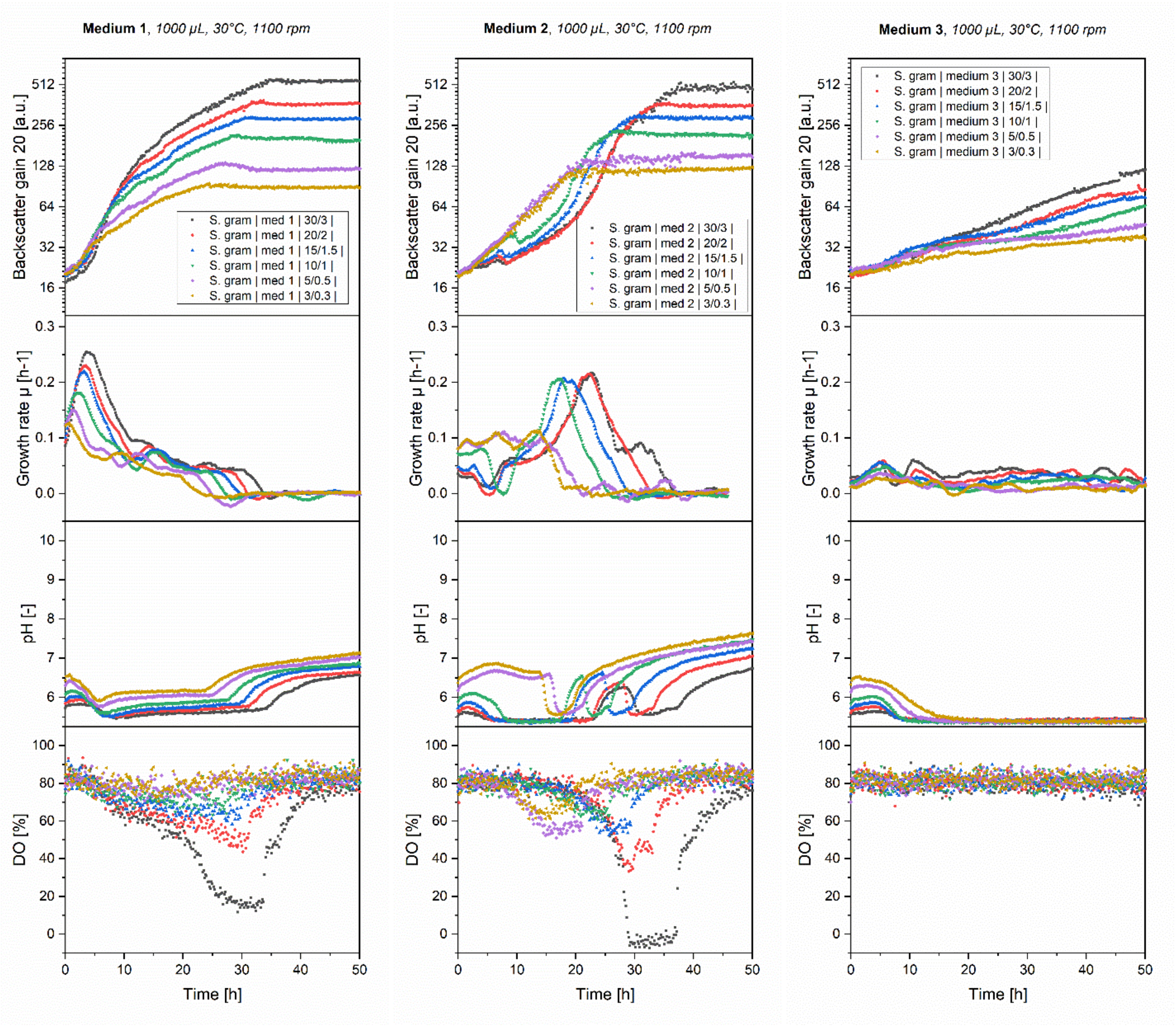

Supplementary Figure 6 Online growth profiles (backscatter, growth rates, pH and DO) for *S. graminicola* in medium 1 (left), medium 2 (middle) and medium 3 (right) with varying medium concentrations from 3 to 30 g L^{-1} glucose. Higher substrate concentrations led to higher final backscatter, slightly increasing growth rates and lower dissolved oxygen levels, i.e. higher oxygen demand.

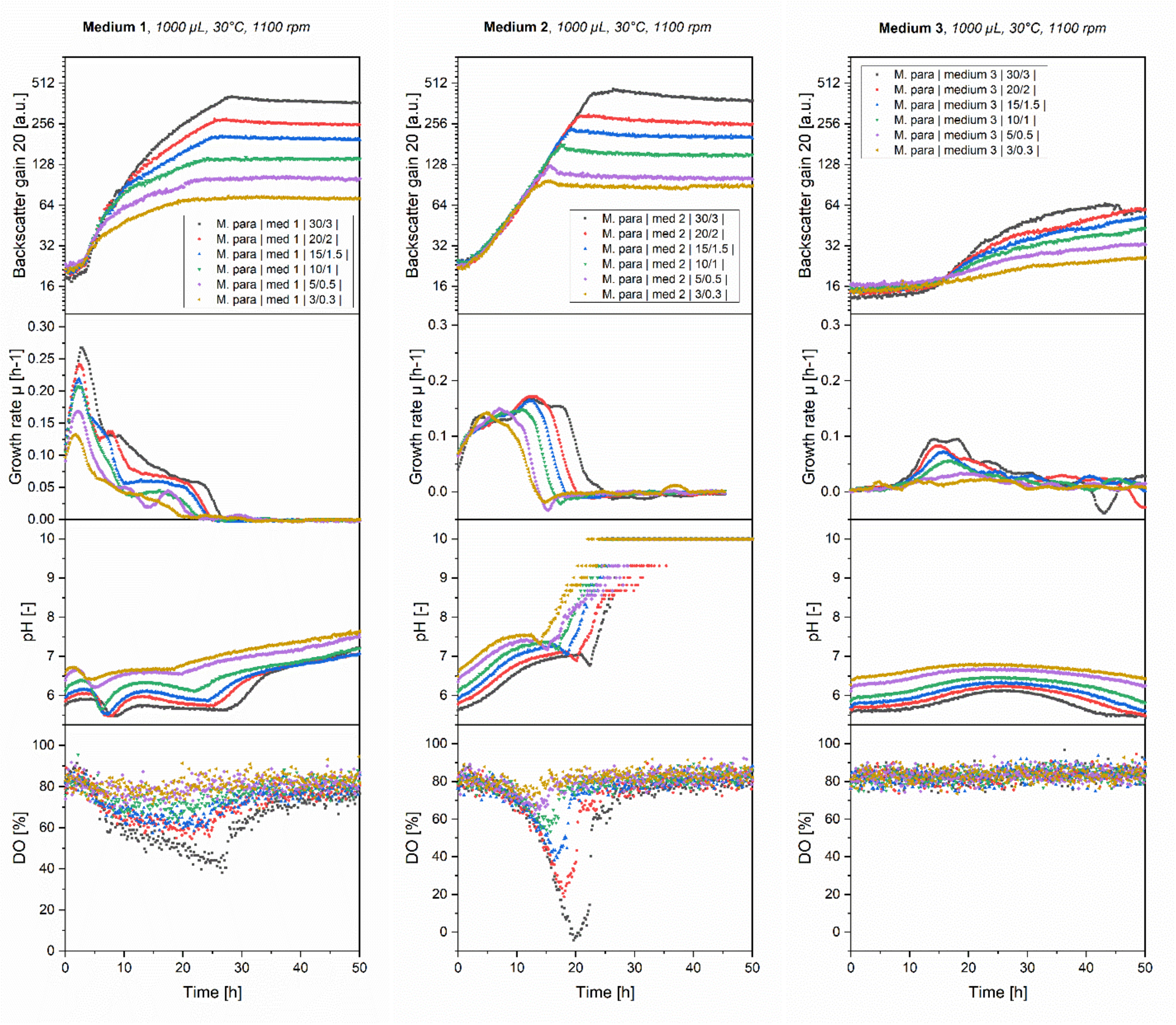

Supplementary Figure 7 Online growth profiles (backscatter, growth rates, pH and DO) for *M. parantarcticus* in medium 1 (left), medium 2 (middle) and medium 3 (right) with varying medium concentrations from 3 to 30 g L^{-1} glucose. Higher substrate concentrations led to higher final backscatter, slightly increasing growth rates and lower dissolved oxygen levels, i.e. higher oxygen demand.

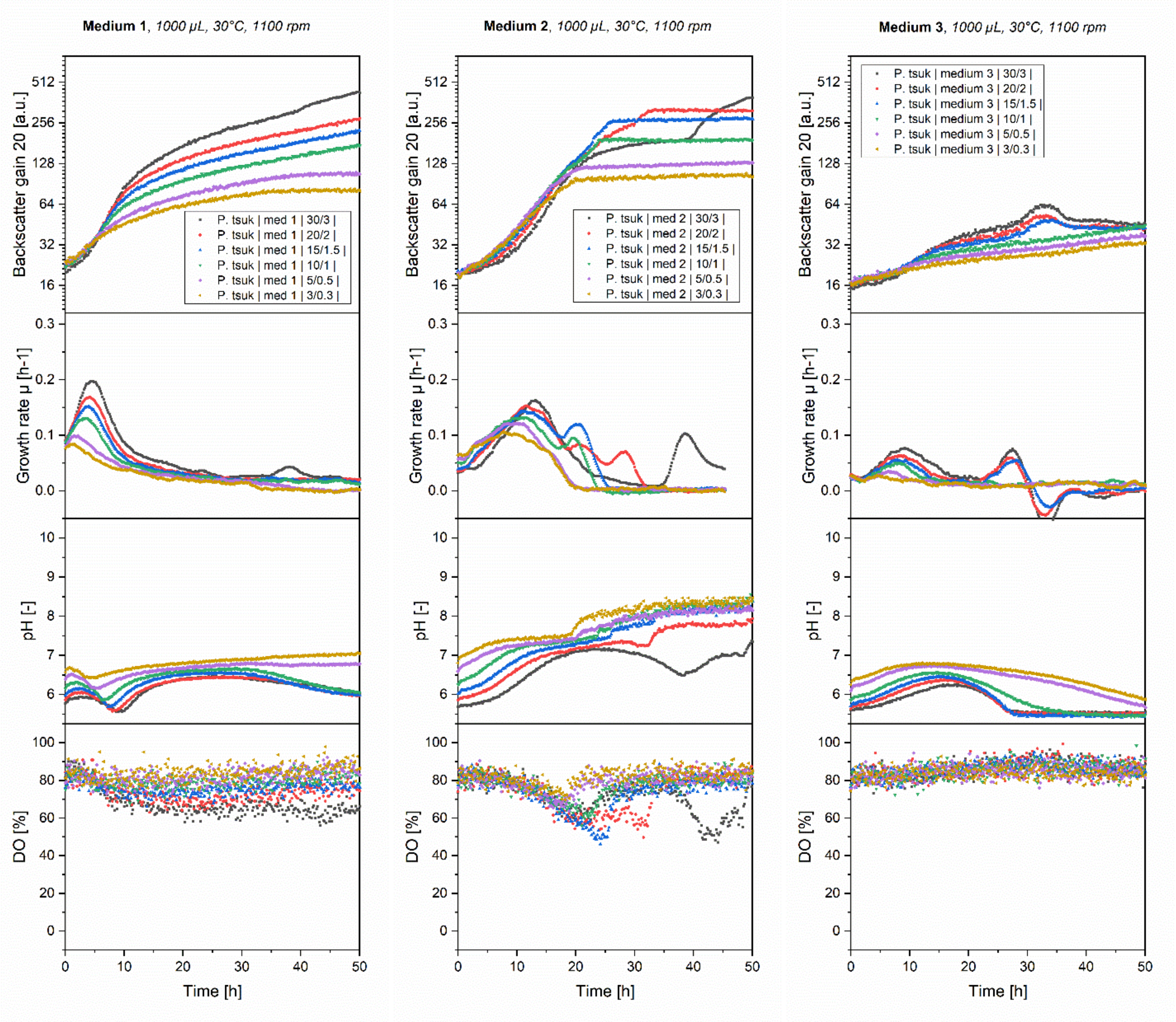

Supplementary Figure 8 Online growth profiles (backscatter, growth rates, pH and DO) for *P. tsukubaensis* in medium 1 (left), medium 2 (middle) and medium 3 (right) with varying medium concentrations from 3 to 30 g L^{-1} glucose. Higher substrate concentrations led to higher final backscatter, slightly increasing growth rates and lower dissolved oxygen levels, i.e. higher oxygen demand.

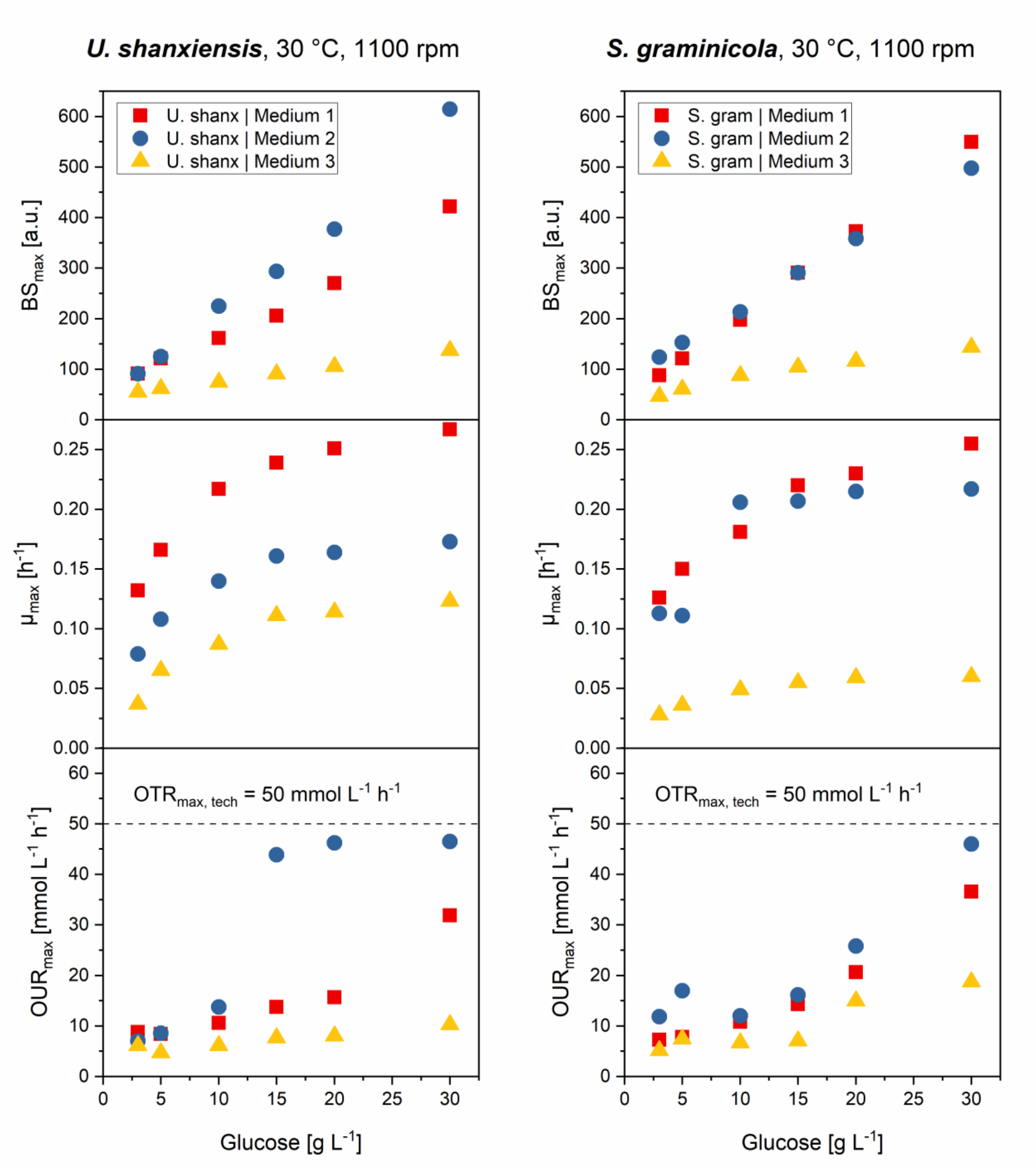

Supplementary Figure 9 Comparison of max. final backscatter BS_{max}, max. specific growth rate μ_{max} and max. oxygen uptake rates OUR_{max} for *U. shanxiensis* (left) and *S. graminicola* (right) in the three media with increasing medium concentrations from 3 to 30 g L^{-1} glucose equivalent. BS_{max} values were comparable between medium 1 and 2, and increased proportionally with medium concentration. Maximum growth rates μ_{max} increased until the point of oxygen limitation. Oxygen limitation occurred when the OUR_{max} values were equal to the OTR_{max}. The respective medium concentration that led to oxygen limitation was different for each species and medium.

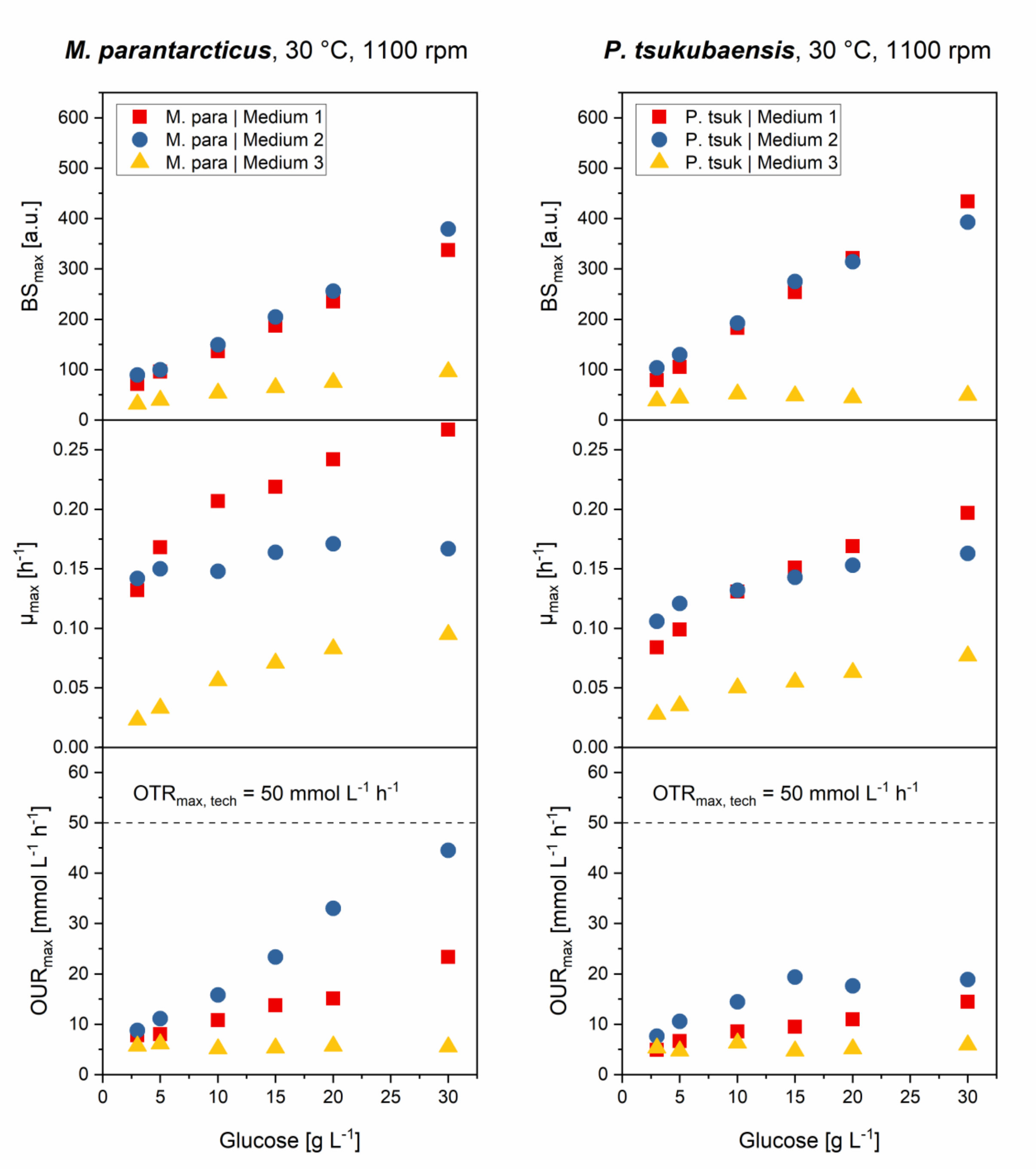

Supplementary Figure 10 Comparison of max. final backscatter BS_{max}, max. specific growth rate μ_{max} and max. oxygen uptake rates OUR_{max} for *M. parantarcticus* (left) and *P. tsukubanesis* (right) in the three media with increasing medium concentrations from 3 to 30 g L^{-1} glucose equivalent. BS_{max} values were comparable between medium 1 and 2, and increased proportionally with medium concentration. Maximum growth rates μ_{max} increased until the point of oxygen limitation. Oxygen limitation occurred when the OUR_{max} values were equal to the OTR_{max}. The respective medium concentration that led to oxygen limitation was different for each species and medium.

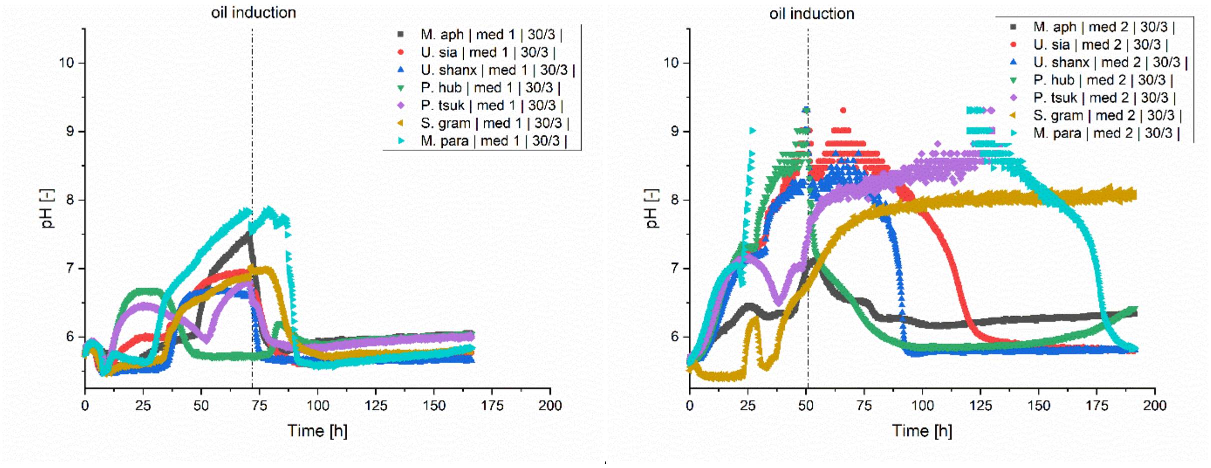

Supplementary Figure 11 Online pH values during growth and production phase (after oil addition) for all seven *Ustilaginaceae* species in medium 1 (left) and medium 2 (right) with an initial concentration of 30 g L^{-1} glucose. Growth phase lasted for 72 h in medium 1 and 48 h in medium 2, before oil was added.

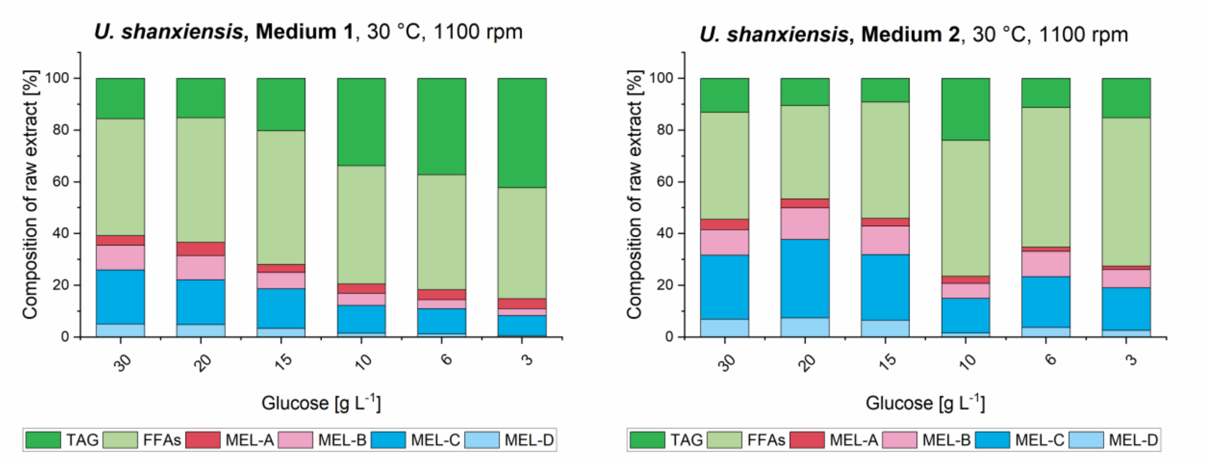

Supplementary Figure 12 Composition of the organic extracts from *U. shanxiensis* cultures with increasing initial concentrations in medium 1 (left) and medium 2 (right), taken from the microtiter plates after MEL production was induced with 8 % v/v rapeseed oil. Relative amounts of unconsumed triglycerides (TAG), free fatty acids (FFAs) and the different MEL variants are shown.

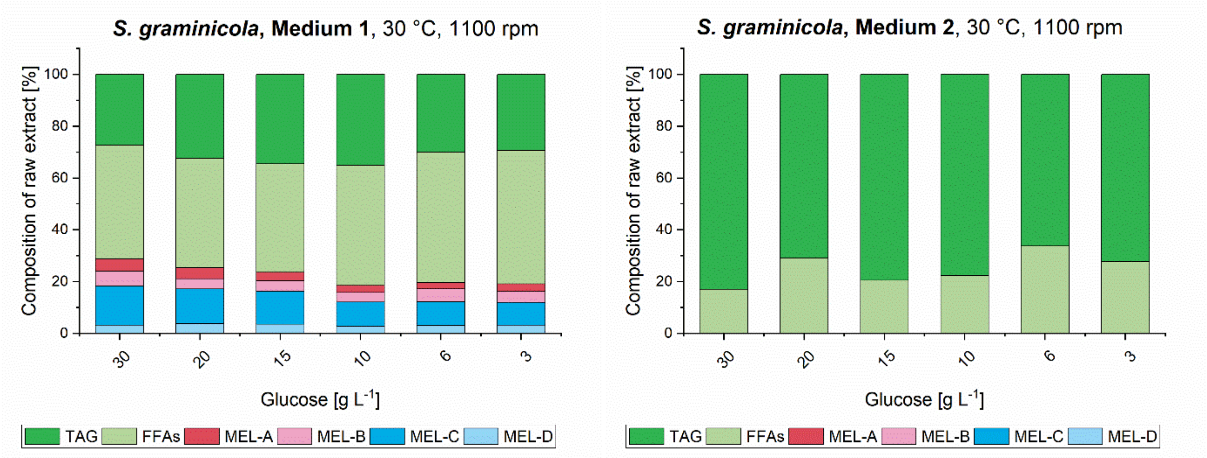

Supplementary Figure 13 Composition of the organic extracts from *S. graminicola* cultures with increasing initial concentrations in medium 1 (left) and medium 2 (right), taken from the microtiter plates after MEL production was induced with 8 % v/v rapeseed oil. Relative amounts of unconsumed triglycerides (TAG), free fatty acids (FFAs) and the different MEL variants are shown.

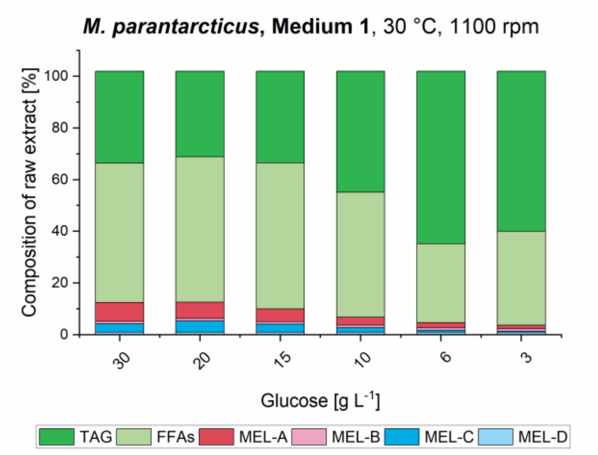

Supplementary Figure 14 Composition of the organic extracts from *M. parantarcticus* cultures with increasing initial concentrations in medium 1, taken from the microtiter plates after MEL production was induced with 8 % v/v rapeseed oil. Relative amounts of unconsumed triglycerides (TAG), free fatty acids (FFAs) and the different MEL variants are shown.

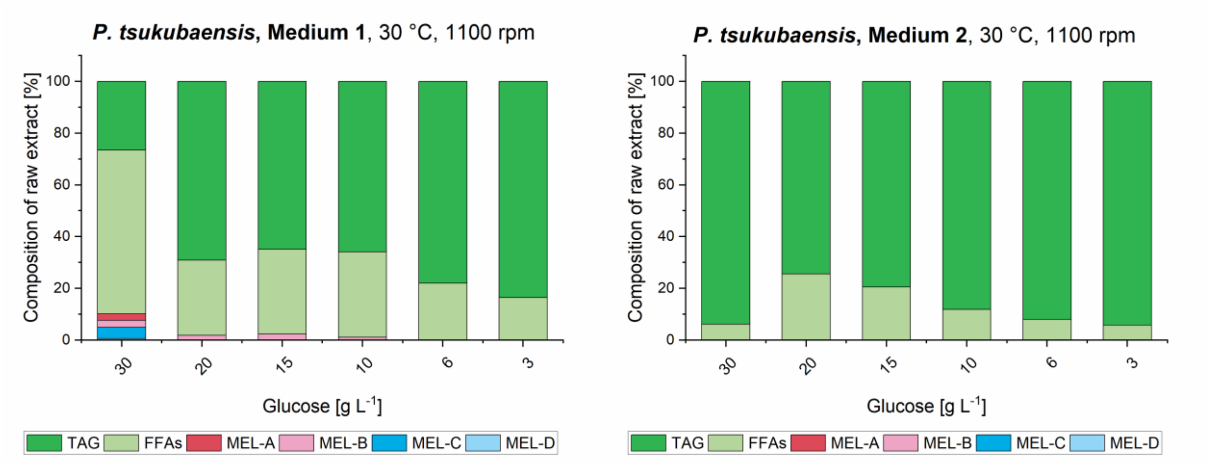

Supplementary Figure 15 Composition of the organic extracts from *P. tsukubaensis* cultures with increasing initial concentrations in medium 1 (left) and medium 2 (right), taken from the microtiter plates after MEL production was induced with 8 % v/v rapeseed oil. Relative amounts of unconsumed triglycerides (TAG), free fatty acids (FFAs) and the different MEL variants are shown.
